# MFGE8 in exosomes derived from mesenchymal stem cells prevents esophageal stricture after endoscopic submucosal dissection in pigs

**DOI:** 10.1186/s12951-024-02429-0

**Published:** 2024-04-01

**Authors:** Huasheng Lai, Hon-Chi Yip, Yu Gong, Kai-Fung Chan, Kevin Kai-Chung Leung, Melissa Shannon Chan, Xianfeng Xia, Philip Wai-Yan Chiu

**Affiliations:** 1 Department of Gastroenterology and Hepatology, Guangzhou Key Laboratory of Digestive Diseases, Guangzhou Digestive Disease Center, Guangzhou First People’s Hospital, School of Medicine, South China University of Technology, Guangzhou, 510180 People’s Republic of China; 2 Department of Surgery and State Key Laboratory of Digestive Disease, Institute of Digestive Disease, The Chinese University of Hong Kong, Hong Kong SAR, 999077 People’s Republic of China; 3 Department of Endoscopy, State Key Laboratory of Oncology in South China, Guangdong Provincial Clinical Research Center for Cancer, Sun Yat-sen University Cancer Center, Guangzhou, 510060 People’s Republic of China; 4 Department of Biomedical Engineering, The Chinese University of Hong Kong, Hong Kong SAR, 999077 People’s Republic of China; 5 Chow Yuk Ho Technology Center for Innovative Medicine, The Chinese University of Hong Kong, Hong Kong SAR, 999077 People’s Republic of China

**Keywords:** Mesenchymal stem cells, Exosomes, MFGE8, Endoscopic submucosal dissection, Esophageal stricture

## Abstract

**Background:**

Endoscopic submucosal dissection (ESD) is the current standard treatment for early-stage esophageal neoplasms. However, the postoperative esophageal stricture after extensive mucosal dissection remains a severe challenge with limited effective treatments available. In this study, we introduced a chitosan/gelatin (ChGel) sponge encapsulating the adipose mesenchymal stem cells (ADMSCs)-derived exosomes (ChGel^MSC−Exo^) for the prevention of esophageal stenosis after ESD in a porcine model.

**Results:**

Pigs were randomly assigned into (1) ChGel^MSC−Exo^ treatment group, (2) ChGel^PBS^ group, and (3) the controls. Exosome treatments were applied immediately on the day after ESD as well as on day 7. Exosome components crucial for wound healing were investigated by liquid chromatography-tandem mass spectrometry (LC–MS/MS) and small RNA sequencing. ChGel^MSC−Exo^ treatment significantly reduced mucosal contraction on day 21, with less fiber accumulation and inflammatory infiltration, and enhanced angiogenesis when compared with the control and ChGel^PBS^ groups. The anti-fibrotic effects following MSC-Exo treatment were further found to be associated with the anti-inflammatory M2 polarization of the resident macrophages, especially within the M2b subset characterized by the reduced TGFβ1 secretion, which sufficiently inhibited inflammation and prevented the activation of myofibroblast with less collagen production at the early stage after ESD. Moreover, the abundant expression of exosomal MFGE8 was identified to be involved in the transition of the M2b-macrophage subset through the activation of MFGE8/STAT3/Arg1 axis.

**Conclusions:**

Our study demonstrates that exosomal MFGE8 significantly promotes the polarization of the M2b-macrophage subset, consequently reducing collagen deposition. These findings suggest a promising potential for MSC-Exo therapy in preventing the development of esophageal stricture after near-circumferential ESD.

**Graphical Abstract:**

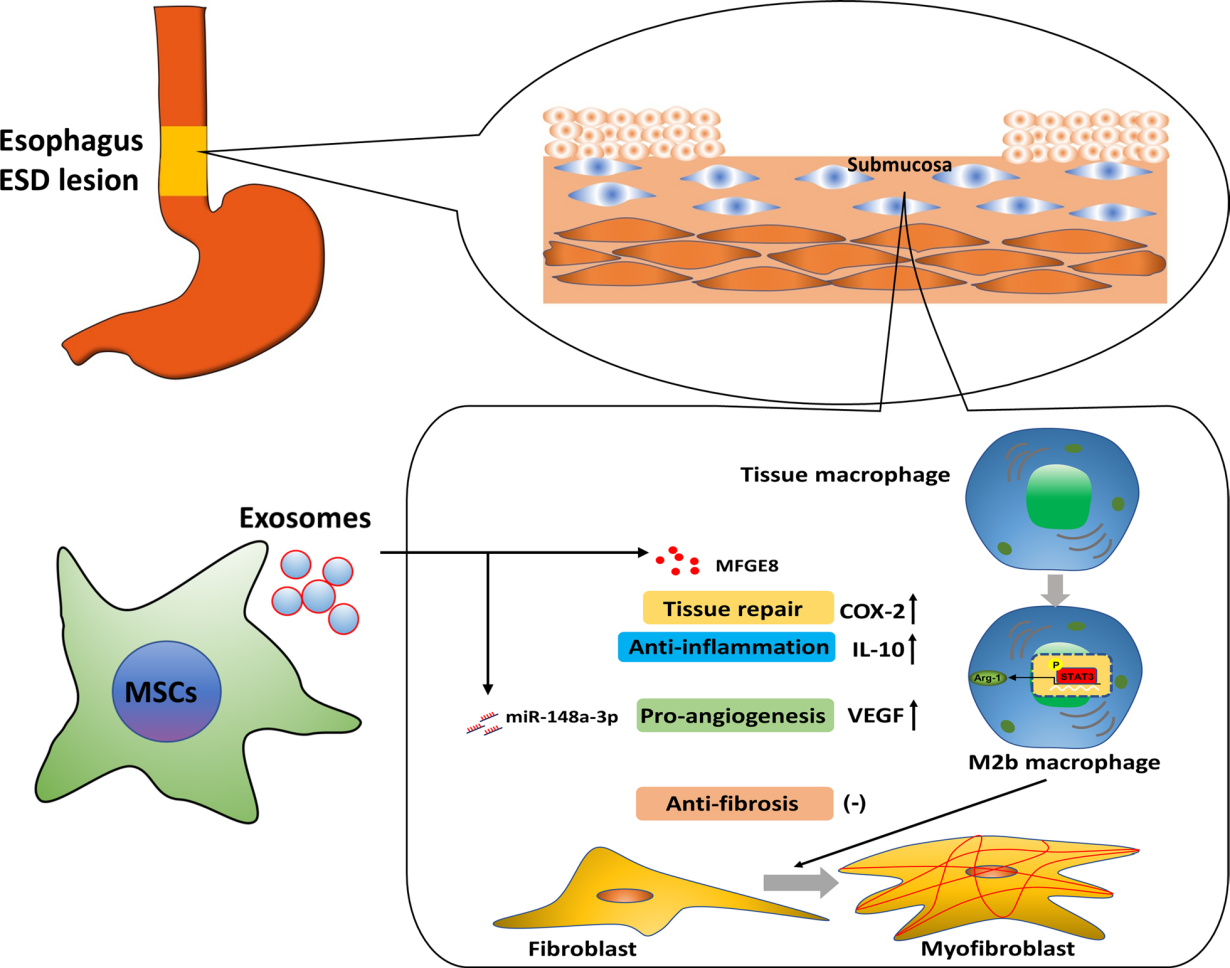

**Supplementary Information:**

The online version contains supplementary material available at 10.1186/s12951-024-02429-0.

## Background

An estimated 604,100 new cases of esophageal cancer occurred in 2020 worldwide [1]. Endoscopic submucosal dissection (ESD) has been accepted as the standard treatment procedure for superficial esophageal neoplasms [2, 3]. However, one of the major complications after extensive endoscopic resection is postoperative esophageal stricture, leading to significant morbidity for patients. Ono et. al reported that nearly 90% of patients with lesions of more than three-quarters of the luminal circumference suffered postoperative esophageal stricture after ESD [4]. These patients were treated by either repeated endoscopic balloon dilation or temporary stent [5], but both modalities possess potential risks including esophageal perforation and mediastinitis [6]. The other modalities for preventing esophageal stricture after ESD include systemic administration or locoregional injection of steroids (e.g. Triamcinolone), reducing the incidence of stricture by approximately 30 to 50% [7–9], but the evidence of efficacy has not so far been sufficient to generate a consensus [10]. Frequent administrations of steroids may also induce adverse events such as immune suppression, diabetes, peptic ulcerations, osteoporosis, and the susceptibility to infection.^7^

Adipose-derived mesenchymal stem cells (ADMSCs) exhibit the capacity for multilineage differentiation and robust paracrine activity by secreting cytokines, chemokines and trophic factors [11], indicating the promising prospects for tissue regeneration [12, 13]. Our previous studies demonstrated that ADMSCs significantly promoted healing of the acid/NSAID-induced gastric mucosal injury, primarily through their paracrine mechanisms [14, 15]. Exosomes are the extracellular vesicles responsible for the cell-to-cell communication through transferring encapsulating protein and functional RNAs (mRNA, miRNA) [16]. Studies have shown that exosomes can mediate the normal physiology and show promise in treating myocardial infarction, diabetes, and hepatic and renal fibrosis [17–19]. A recent study has demonstrated that the conditioned medium from MSCs relieved mucosal stricture rate by approximately 24–28% after ESD [20], although the potential bioactive compounds responsible for the therapeutic effect remained unclear. In this study, we aimed to investigate the feasibility and efficacy of MSC-derived exosomes in preventing esophageal stricture after ESD in porcine models. A porous chitosan/gelatin sponge scaffold (ChGel sponge) was introduced as a carrier to endoscopically deliver the encapsulated exosomes to the esophageal mucosal defect after ESD.

## Results

### Characterization of ADMSCs, MSC-Exo and exosomes encapsulated ChGel sponges

ADMSCs were isolated from abdominal subcutaneous fat tissues of pig donors (Additional file 1: Fig. S1A) [21]. Flow cytometry showed that cells were negative for CD45 (hematopoietic stem cell-related marker) but positive for CD90, CD44, CD105, and CD29 (MSC-related markers) (Additional file 1: Fig. S1B). Experiments in vitro confirmed their ability of multilineage differentiation for chondrogenesis, adipogenesis and osteogenesis (Additional file 1: Fig. S1C).

**Fig. 1:**
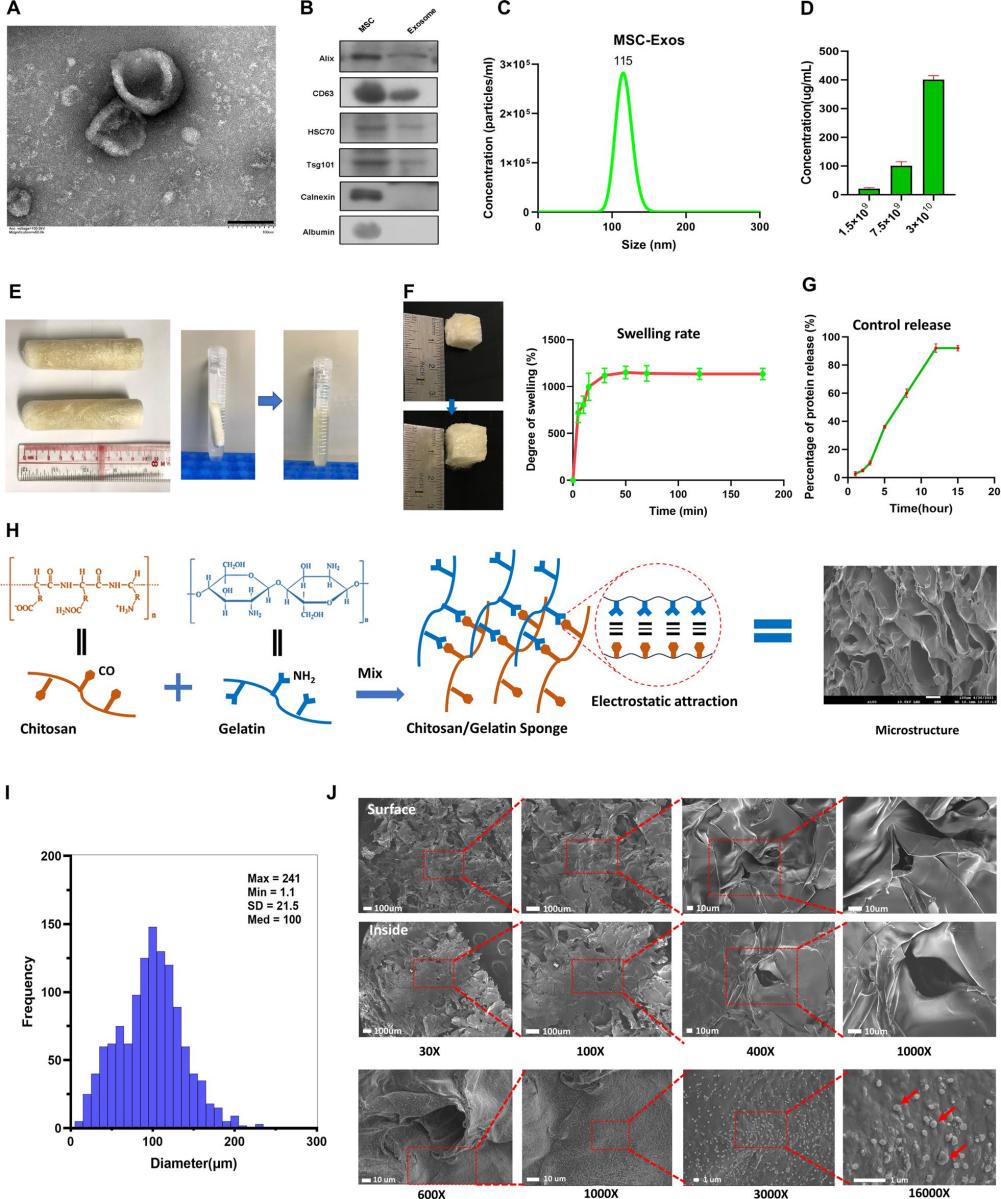
The characterization of MSC-Exo and ChGel sponges. **A** Exosomes captured by transmission electron microscopy (TEM, bar: 100 nm). **B** Exosome specific protein markers including the Alix, CD63, HSC70 and Tsg101, and the negative markers including the Calnexin and Albumin were detected by Western Blot. **C** Size distribution of MSC-Exo on the basis of nanoparticle tracking analysis. **D** Correlation between the quantity of particles and the protein concentration. **E** Morphology and degradability of ChGel sponge. **F** Changes in the volume and weight of the sponge after absorbing PBS. **G** Controlled release test of MSC-Exo from the ChGel sponge scaffold. **H**, **I** Schematic illustration of the putative cross-linked structure and the pore size distribution of ChGel sponge. **J** The surface and inner structure of the cross-linked scaffold identified by SEM, which confirmed the effective encapsulation of MSC-Exo within and on the surface of the sponge scaffold. The red arrows indicated the encapsulated exosomes

ADMSCs were then cultured in exosome-free medium. The conditioned medium (MSC-CM) was collected after 48 h and subjected to differential centrifugation to extract extracellular vesicles (EVs), followed by the characterization with transmission electron microscopy (TEM), nanoparticle tracking analysis (NTA) and western blot. The EVs showed classic cup-shaped morphology resembling that of exosomes (MSC-Exos) (Fig. 1A). They were positive for Alix, CD63, HSC70 and Tsg101 (the exosomal specific markers), but negative for Calnexin and Albumin (Fig. 1B). Evaluation of the size distribution of EVs by NTA revealed a peak at 115 nm (Fig. 1C). The correlation between the quantity of the particles and the concentration was also determined (Fig. 1D).

To construct the functionalized porous ChGel scaffolds, a mixture of chitosan and gelatin liquid was lyophilized to produce sponges. The construction method is illustrated in Additional file 1: Fig. S2 and the detailed protocol was presented in the Methods. We created a cylindrical shaped sponge around 10 cm in length and 2.5 cm in width (Fig. 1E). The physicochemical properties of ChGel scaffolds were then evaluated. The sponge scaffold completely dissolved upon immersion in hydrochloric acid with a pH of 3.0 (Fig. 1E), suggesting that it would undergo rapid degradation when exposed to the gastric acid. Importantly, this degradation process ensures the scaffold will not induce any significant risk of gastrointestinal obstruction when it is pushed into the stomach by food consumption. In addition, the ChGel sponge swelled to around twice its original volume (Fig. 1F) and weighed over 10 times its original weight after absorbing PBS solution for 15 min, suggesting the good property of absorbing the diluted exosomes. The controlled release test also indicated that the encapsulated exosomes could be gradually released from the sponge, with the equilibrium reaching at 90% by 12 h (Fig. 1G). Moreover, scanning electron microscopy (SEM) revealed the ChGel sponge scaffold with a composite network characterized by the reticular and irregularly cross-linked porous structures (Fig. 1H), probably arising from the cross-linking mechanism facilitated by the electrostatic attraction between the NH2 group of chitosan and the CO group of gelatin [22]. The median diameter of the pore size was approximately 100 μm, with a range from 1.1 to 241 μm (Fig. 1I). SEM further showed the effective encapsulation of MSC-Exo within and on the surface of the sponge scaffold (Fig. 1J).

To examine the cytocompatibility of the materials, scaffold components were coated in a 6-well cell plate and afterwards seeded with RAW 264.7 and 3T3-L1 cells. The live/dead cell staining revealed that around 84.1% of 3T3-L1 and 92.5% of RAW 264.7 cells cultured with ChGel remained viable after 48 h respectively, which were comparable to that of the PBS control (Additional file 1: Fig. S3A, B).

### Effect of exosome-encapsulated sponges on esophageal stricture prevention in a porcine model

**Fig. 2:**
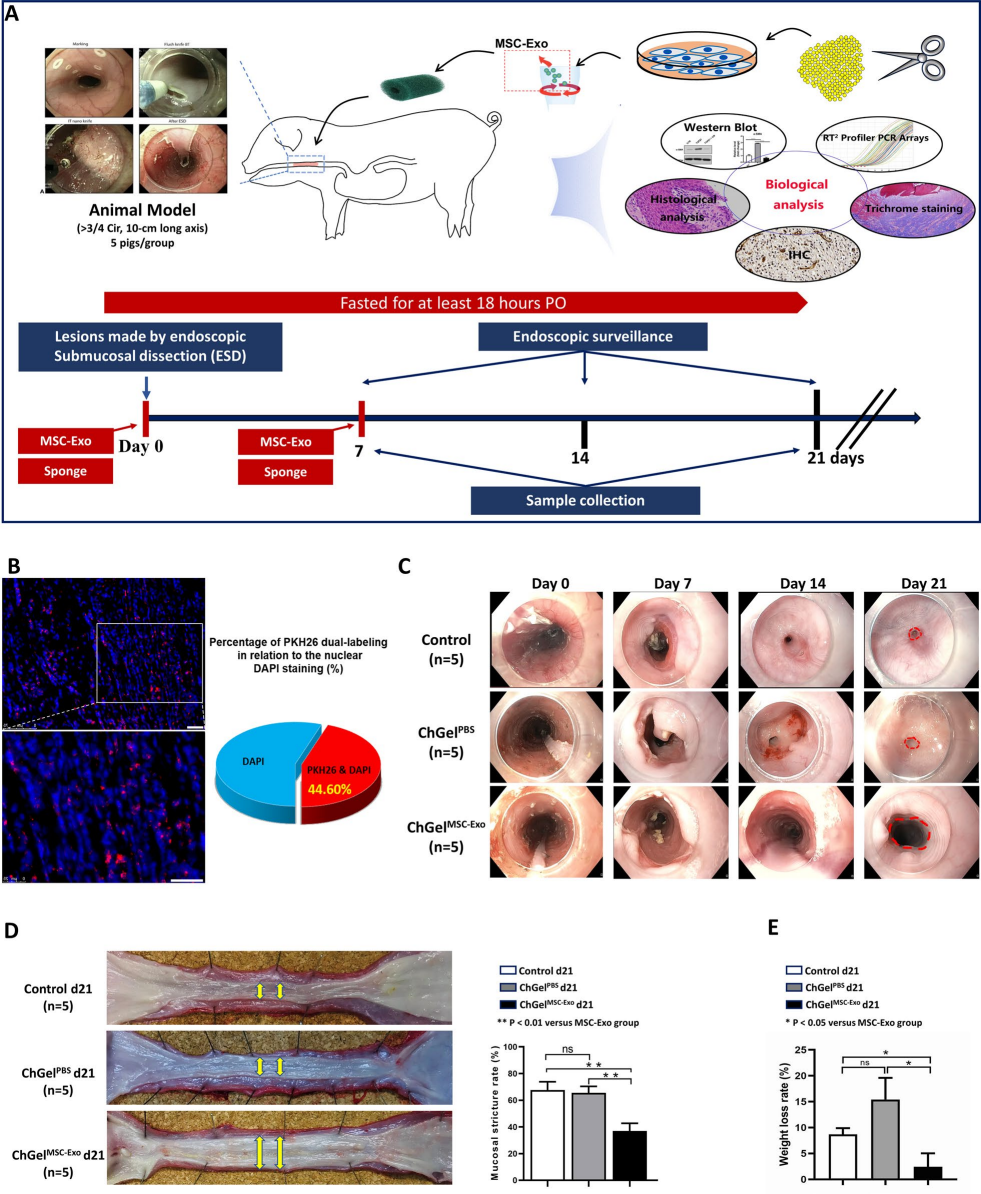
A porcine model of esophageal stricture is induced by near-circumferential ESD. **A** Proposal for investigating the feasibility and effectiveness of the sponge scaffold encapsulated with the adipose-derived mesenchymal stem cells (ADMSCs)-derived exosomes in preventing esophageal stricture in pigs. **B** Fluorescent microscopy showed the fluorescence signals of PKH26 labelled MSC-Exo at the submucosa of esophagus (Scale bars, 100 μm). Quantitative analysis showed approximately 44.6% of the tissue cells took in the released MSC-Exo at the surgical site. **C** Endoscopic surveillance on day 7, 14, and 21 was conducted to evaluate the esophageal stricture in pigs. **D** Comparison of the mucosal stricture rate among three groups. **E** Comparison of the weight loss among three groups. All data are shown as the means ± SEM. Statistical significance was analyzed by one-way ANOVA, followed by a Tukey post hoc analysis. *P < 0.05, **P < 0.01. *PO* pre-operation

To investigate the effect of stricture prevention with MSC-Exo treatment, a near-circumferential endoscopic submucosal dissection (ESD) was performed to remove the large-area esophageal mucosa with 10 cm in length in porcine models. Quality control of esophageal ESD was presented in Table 1. A cylindrical shaped sponge with the same length of 10 cm and width of 2.5 cm was constructed in order to fit and shield the luminal mucosal defect. A total of 24 pigs were recruited and were assigned into the MSC-Exo treatment group (ChGel^MSC−Exo^), the sponge control group (ChGel^PBS^) and the negative control group with no intervention (Control). For the ChGel^MSC−Exo^ group, the ChGel scaffold absorbing exosomes (1 mg) in PBS solution (10 mL) were delivered endoscopically to shield the esophageal wound immediately after ESD (Additional file 2: Movie S1). For the ChGel^PBS^ group, the ChGel sponge absorbing 10 mL PBS solution was applied topically. A second treatment in the ChGel^MSC−Exo^ group and ChGel^PBS^ group was performed on day 7 (Fig. 2A). Pigs remained fasting after ESD while receiving parenteral nutrition (197 kcal/kg/day) for 48 h. Due to the adhesive property of chitosan to the gastrointestinal mucosa, the sponge scaffold was observed to remain in place within the esophageal lumen for 8–12 h while the natural peristaltic movement of the esophagus would eventually push the scaffold into the stomach. To maximize its maintenance in situ, two clips were applied to secure the upper border of the sponge scaffold (Additional file 2: Movie. S1). Furthermore, experiment in vivo showed the presence of intracellular red fluorescence (PKH-26-labled exosomes) in approximately 44.6% of cells at the submucosa layer of the wound on 12 h, confirming the release of the encapsulated MSC-Exo from sponge scaffold and uptake by the tissue cells (Fig. 2B). Endoscopic surveillance was conducted to monitor the esophageal stricture on days 7, 14 and 21. The appetite of animals, food consumption, and activity were observed daily. The body weight of each pig was recorded on day 0 and day 21 respectively. Pigs were sacrificed on day 7 (Control, ChGel^PBS^ and ChGel^MSC−Exo^; *n* = 3/group), and day 21 (Control, ChGel^PBS^ and ChGel^MSC−Exo^; *n* = 5/group) respectively.

**Table 1:** Quality control of esophageal ESD

Parameter	Control group (n = 8)	ChGel^PBS^ group (n = 8)	ChGel^MSC−Exo^ group (n = 8)	*P*
Mean duration (min), mean ± SD	69.3 ± 10.8	73.0 ± 12.5	73.0 ± 9.9	0.74
Total volume for submucosal Injection (mL), mean ± SD	77.1 ± 8.9	74.9 ± 10.7	76.1 ± 11.5	0.91
Major axis of specimen (mm), mean ± SD	53.9 ± 10.1	54.3 ± 6.5	56.0 ± 5.0	0.83
Adverse events				
Perforation rate	0	0	0	NA
Massive bleeding	0	0	0	NA

*ESD* endoscopic submucosal dissection, *SD* standard deviation

Esophageal stricture was observed in both the control and the ChGel^PBS^ group by day 7, with substantial endoscopic manifestations on day 14 and 21 (Fig. 2C). The ChGel^MSC−Exo^ treatment group achieved significantly attenuated esophageal stricture on either days 7, 14 or 21 when compared to the control and ChGel^PBS^ group respectively (Fig. 2C). Macroscopic investigation showed that the esophageal ESD defect in the ChGel^MSC−Exo^ group shrunk by around 37.01% relative to the normal mucosa area on the upper and lower sides, which was significantly lower than that in the control (*vs* 67.71%, *P* < 0.01) and the ChGel^PBS^ groups (*vs* 65.54%, *P* < 0.01) respectively (Fig. 2D). The body weight loss of pigs on day 21 reached 8.71% in the control group and 15.42% in the ChGel^PBS^ group, both of which were significantly higher than that in the ChGel^MSC−Exo^ group (*vs* 2.42% ± 2.60%, *P* = 0.013 and *P* = 0.021, respectively; Fig. 2E). However, no significant difference was found between the ChGel^PBS^ and control groups on the stricture formation (the mucosal stricture rate: 65.54% vs 67.71%, *P* = 0.912; Fig. 2D) as well as on the weight loss (15.42% vs 8.7%; *P* = 0.273; Fig. 2E) on day 21.

### Treatment with ChGel^MSC−Exo^ alleviated fibrosis of the esophageal wound in pigs

**Fig. 3:**
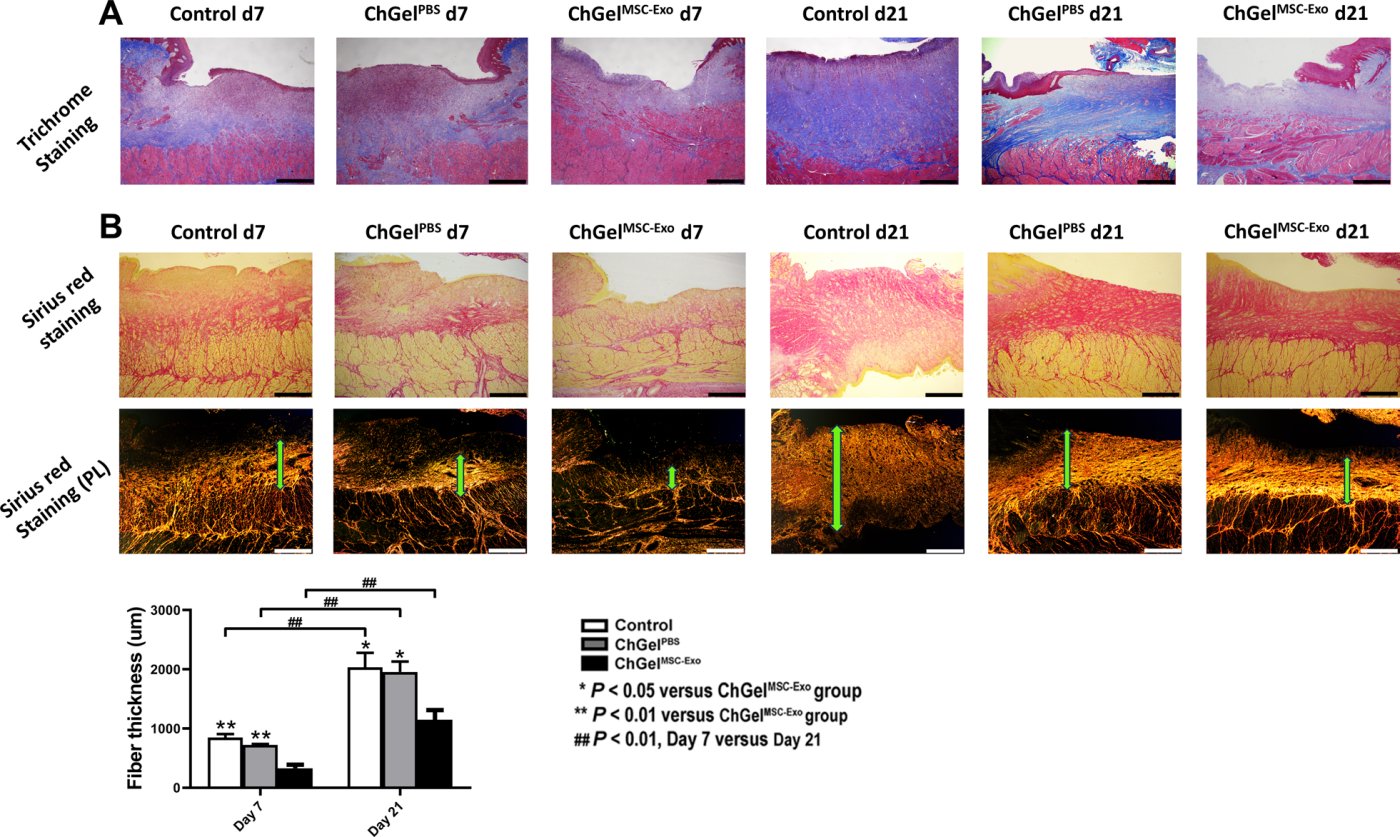
Histological evaluation of the fiber thickness by Masson’s trichrome and Sirius red staining. **A** Masson’s trichrome staining on samples obtained by days 7 and 21. **B** Sirius red staining on samples obtained by days 7 and 21 and examination under polarized light mode. Comparison of the fiber thickness was conducted among three groups as well as between the time points of days 7 and 21. Statistical significance was analyzed by independent samples *t* test or one-way ANOVA followed by a Tukey post hoc analysis between two or multiple groups, respectively. *P < 0.05, **P < 0.01, ## *P* < 0.01. Scale bars, 1 mm. *PL* polarized light mode of microscope

Collagen deposition and fibrosis contribute to the formation of esophageal stricture after ESD [20]. Masson’s trichrome and Sirius red staining demonstrated that the fiber accumulation was more abundant on day 21 than that on day 7 in all groups (*P* < 0.01; Fig. 3A, B). The thickness of collagen fibers was significantly reduced upon ChGel^MSC−Exo^ treatment on day 7 (329 μm ± 119 μm) in comparison with the control group (*vs* 826 μm ± 92 μm, *P* < 0.01; Fig. 3B) and ChGel^PBS^ group *(vs* 721 μm ± 31 μm, *P* < 0.01; Fig. 3B) respectively. Similarly, on day 21, the fiber thickness was significantly lower in the ChGel^MSC−Exo^ group (1146 μm ± 368 μm) than in the control (*vs* 2033 μm ± 547 μm, *P* = 0.015; Fig. 3B) and ChGel^PBS^ groups (*vs* 1956 μm ± 395 μm, *P* = 0.025; Fig. 3B) respectively. There was no significant difference in the level of fiber accumulation between the ChGel^PBS^ and control group on both day 7 and day 21 (Fig. 3B).

**Fig. 4:**
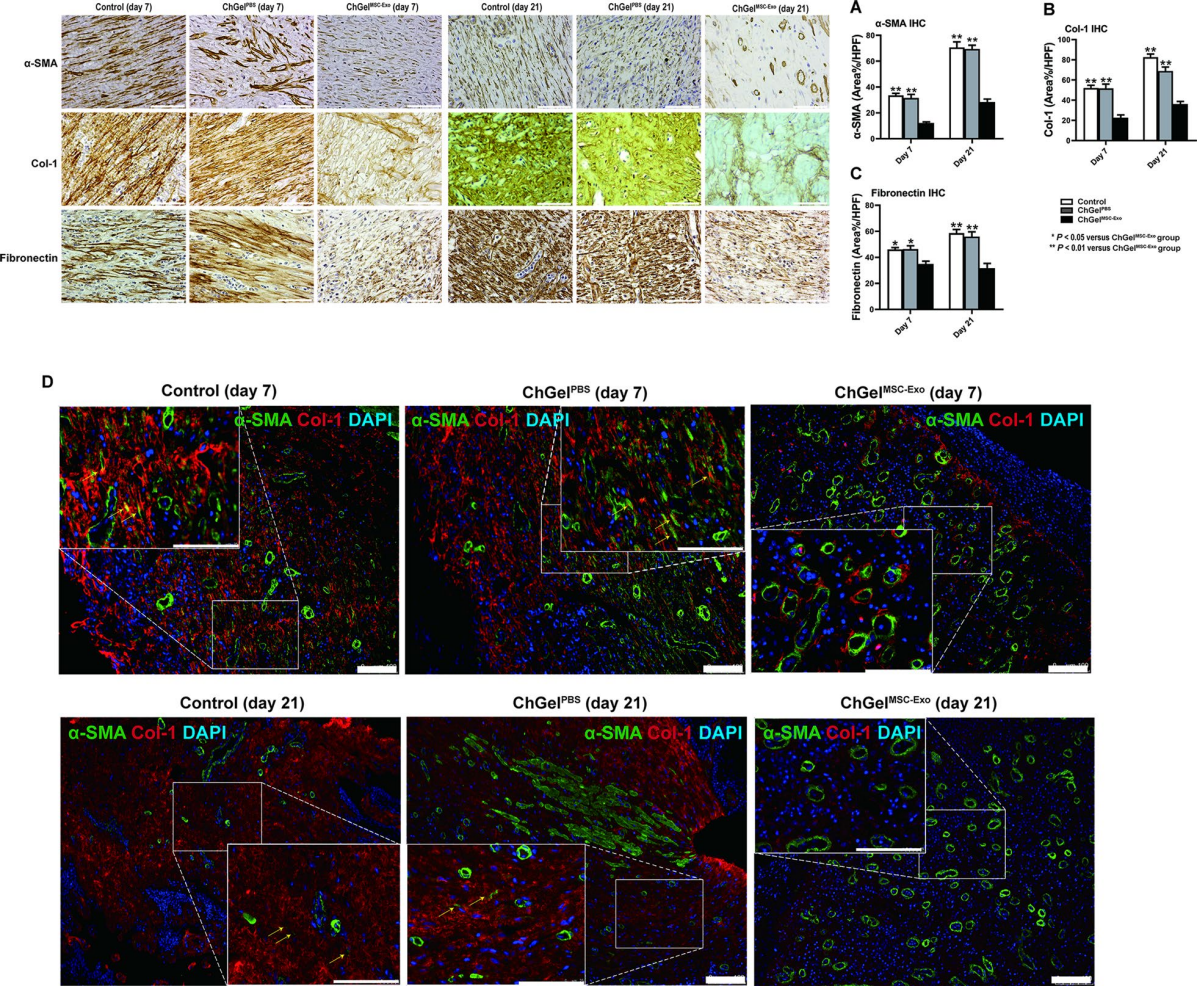
The anti-fibrotic efficiency of ChGel^MSC−Exo^ on days 7 and 21. **A**–**C** The quantification of the IHC results on α-SMA, collagen-I, fibronectin, scale bars, 100 μm. **D** Immunofluorescence double staining for Collagen-I and α-SMA (yellow arrows indicate the myofibroblast cells). Scale bars, 100 μm. All data are shown as the means ± SEM. Statistical significance was analyzed by one-way ANOVA followed by a Tukey post hoc analysis among multiple groups, respectively. *P < 0.05, **P < 0.01. Pigs, *n* = 3 (day 7); *n* = 5 (day 21)

To investigate the anti-fibrotic role of ChGel^MSC−Exo^, histology and western blot were conducted to identify the expression of fibrotic-related proteins on the tissue samples of esophagus. The ChGel^MSC−Exo^ group demonstrated more significant down-regulation of α-SMA, collagen-I than the control and ChGel^PBS^ groups on day 7 with IHC (α-SMA:* P* < 0.01 and *P* < 0.01 respectively, Fig. 4A; Collagen-I: *P* < 0.01 and *P* < 0.01 respectively, Fig. 4B) and western blot (α-SMA:* P* < 0.05 and *P* < 0.05 respectively, Additional file 1: Fig. S4A; Collagen-I: *P* < 0.05 and *P* < 0.05 respectively, Additional file 1: Fig. S4B). The α-SMA and Collagen I levels were also lower in the ChGel^MSC−Exo^ group than in the control and ChGel^PBS^ groups respectively on day 14 and day 21 respectively (Additional file 1: Fig. S4A, B), indicating that ChGel^MSC−Exo^ treatment was capable of suppressing the fibrotic progression in the esophagus after near-circumference ESD at both early and late time periods. Fibronectin is another pro-fibrotic marker [23]. IHC analysis demonstrated that ChGel^MSC−Exo^ treatment significantly inhibited the expression of fibronectin on both day 7 (*P* < 0.05 and *P* < 0.05 respectively, Fig. 4C) and day 21 (*P* < 0.01 and *P* < 0.01 respectively, Fig. 4C) when compared with the control and ChGel^PBS^ groups respectively.

Myofibroblasts are known to be the main cells contributing to collagen production and fibrosis during extracellular matrix remodeling [20]. p-Smad2 is a positive prognostic marker for myofibroblast differentiation [24]. p-Smad2 level significantly reduced after ChGel^MSC−Exo^ treatment on days 7, 14 and 21 (Additional file 1: Fig. S4C) compared with the control and ChGel^PBS^ groups respectively. Double immunofluorescence further identified the collagen-I^+^/α-SMA^+^ myofibroblasts at the submucosa. On day 7 and day 21, less myofibroblast activation in the ChGel^MSC−Exo^ group was observed than that in the control and ChGel^PBS^ groups respectively (Fig. 4D).

**Fig. 5:**
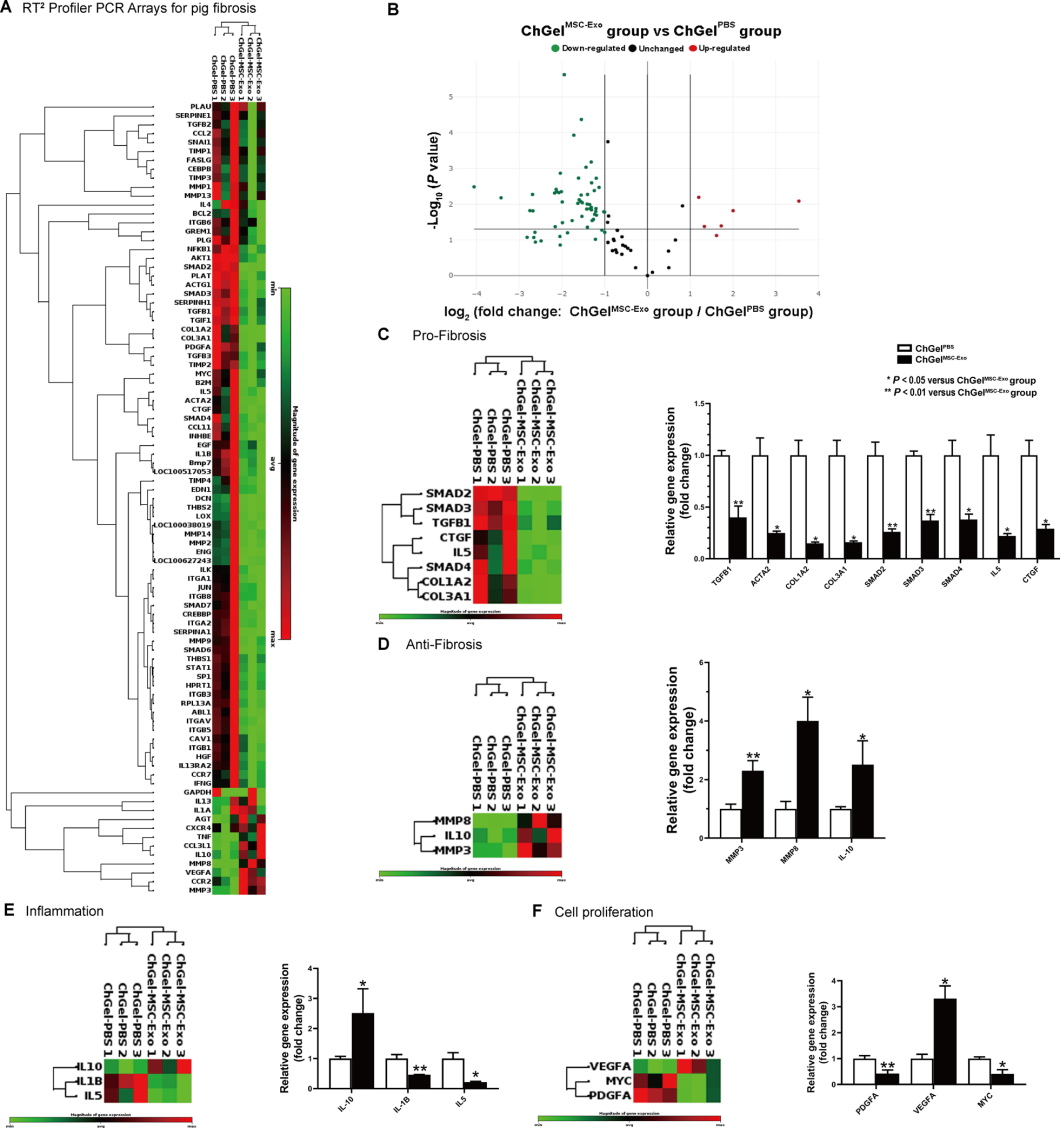
Treatment with MSC-Exo down-regulates genes related to fibrosis in tissue obtained at day 7. **A** Heatmap of the expression of 84 genes in the ChGel^MSC−Exo^ and ChGel^PBS^ group (*n* = 3 per group, black represents the mean magnitude of gene expression, the brightest red and brightest green indicates the maximum and minimum magnitude respectively). **B** Volcano plot of the differentially regulated genes between the ChGel^MSC−Exo^ and ChGel^PBS^ group. **C**–**F** Heatmap of differentially regulated genes involved in the pro-fibrosis, anti-fibrosis, inflammation and cell proliferation. Please refer to Additional file 1: Table S2 for the full gene names. Pigs, *n* = 6. All data are shown as the means ± SEM. Statistical significance was analyzed by independent samples *t* test between two groups, respectively. *P < 0.05, **P < 0.01

Profiling analysis was conducted to investigate the regulation of genes related to fibrosis after ChGel^MSC−Exo^ treatment. RT^2^ Profiler PCR array detected the expression of 84 genes (Additional file 1: Table S2) related to fibrosis and 5 housekeeping genes in the esophageal samples obtained on day 7 (Fig. 5A). A total of 54 genes were significantly down-regulated and 6 genes were up-regulated after ChGel^MSC−Exo^ treatment compared with the ChGel^PBS^ group (Fig. 5B). Specifically, a significant fold change in the gene expression related to pro-fibrosis [*TGFB1*, *ACTA2* (α-SMA), *COL1A2* (Collagen, type I, alpha 2), *COL3A1* (Collagen, type III, alpha 1)*, SMAD2* (SMAD family member 2), *SMAD3*, *SMAD4*, *IL5, CTGF* (connective tissue growth factor)], anti-fibrosis [*MMP3* (matrix metalloproteinase 3), *MMP8*, *IL10*], the functions of inflammation (*IL10*, *IL-1B*, *IL5*) and cell proliferation {*PDGFA* (Platelet-derived growth factor alpha polypeptide)*, VEGFA, MYC* [V-myc myelocytomatosis viral oncogene homolog (avian)]} were shown in Fig. 5C–F respectively.

### Treatment with ChGel^MSC−Exo^ alleviated inflammation, promoted angiogenesis and re-epithelization after esophageal ESD in pigs

Healing of the artificial ulcer after ESD involves the processes of neovascularization, cell migration and epithelial regeneration (re-epithelization), anti-inflammation and ECM remodeling [25]. Our previous study showed that ADMSC-derived conditioned medium enhanced angiogenesis and reduced inflammation of gastric wounds after ESD [14]. We therefore hypothesized that these cellular cascades might also participate to promote healing of esophageal wound after ChGel^MSC−Exo^ treatment.

**Fig. 6:**
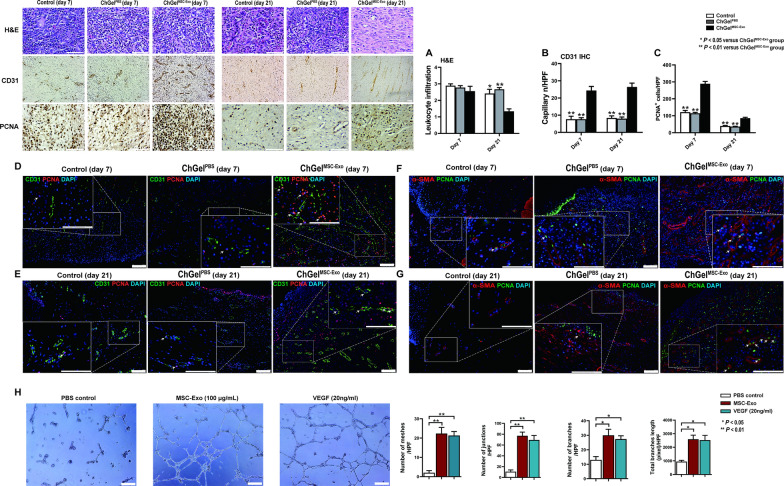
Treatment with MSC-Exo alleviates inflammation, induces the angiogenesis and cellular proliferation. **A** Hematoxylin & eosin (H&E) staining and scoring in terms of leukocyte infiltration. **B**, **C** The quantification of the IHC on CD31 and PCNA on days 7 and 21 respectively; **D**–**G** immunofluorescence double staining for CD31/PCNA (white arrows indicate the endothelial/endothelial progenitor cells) and α-SMA/PCNA (white arrows indicate the vascular smooth muscle cells). Pigs, *n* = 3 (day 7); *n* = 5 (day 21). **H** Representative photographs of tube formation of human microvascular endothelial cells (HMVECs), which were exposured to phosphate-buffered saline (PBS), MSC-Exo (100 μg/mL), or VEGF (20 ng/mL). The number of meshes, junctions, branches formed per high-power field (HPF), and the total length of branches per HPF were calculated by ImageJ software. All data are shown as the means ± SEM. Statistical significance was analyzed by one-way ANOVA followed by a Tukey post hoc analysis among multiple groups, respectively. *P < 0.05, **P < 0.01. Scale bars, 100 μm

We examined the leukocyte infiltration on H&E staining slides to determine the severity of inflammation. Results showed that leukocyte infiltration in esophageal wound obtained on day 7 was similar among the control, ChGel^PBS^ and ChGel^MSC−Exo^ groups (Fig. 6A). On day 21, however, the scoring of leukocyte infiltration was significantly lower in the ChGel^MSC−Exo^ group than the control and ChGel^PBS^ groups (*P* = 0.03 and *P* < 0.01 respectively; Fig. 6A). Expression of interleukin 10 (IL-10) after ChGel^MSC−Exo^ treatment was significantly higher than that in the control and ChGel^PBS^ groups respectively (*P* < 0.01 and *P* < 0.01 on day 7 respectively; Additional file 1: Fig. S4D; *P* < 0.01 and *P* < 0.05 on day 14 respectively, Additional file 1: Fig. S4D), while it gradually decreased to the level comparable to the control and ChGel^PBS^ groups on day 21 (*P* = 0.027 and *P* = 0.044; Additional file 1: Fig. S4D). Meanwhile, the level of Interleukin 1β (IL-1β) in wound samples was significantly reduced in the ChGel^MSC−Exo^ group on day 7 (*vs* control group, *P* = 0.0045 and *vs* ChGel^PBS^ group, *P* = 0.0075, respectively; Additional file 1: Fig. S4E) and day 14 respectively (*vs* control group, *P* = 0.0199; Additional file 1: Fig. S4E), while no significant difference was observed among the groups on day 21 (Additional file 1: Fig. S4E). Western blot showed a significant increase in VEGF protein level in the ChGel^MSC−Exo^ group compared with the control and ChGel^PBS^ groups on day 7 (*P* = 0.0013 and *P* < 0.001, respectively; Additional file 1: Fig. S4F) and day 14 respectively (*P* = 0.0107 and *P* = 0.0035, respectively; Additional file 1: Fig. S4F). On day 21, however, there was no significant difference among the three groups (Additional file 1: Fig. S4F).

To further evaluate the effect of ChGel^MSC−Exo^ on angiogenesis, we examined the capillary density in the wound margin by immunostaining with CD31. Results showed that ChGel^MSC−Exo^ promoted the formation of new capillary vessels. The density of CD31^+^ capillaries was significantly higher in the ChGel^MSC−Exo^ group than the control and ChGel^PBS^ group respectively on both day 7 (*P* < 0.01 and *P* < 0.01, respectively; Fig. 6B) and day 21 (*P* < 0.01 and *P* < 0.01, respectively; Fig. 6B).

The proliferating cell nuclear antigen (PCNA) is a proliferation marker of the G1/S phase. We found that the number of PCNA^+^ cells per field around the wound margin was significantly higher in the ChGel^MSC−Exo^ group than the control (*P* < 0.01; Fig. 6C) and ChGel^PBS^ groups both on day 7 (*P* < 0.01; Fig. 6C) and day 21 (*vs* control, *P* < 0.01 and *vs* ChGel^PBS^ group, *P* < 0.01, respectively; Fig. 6C). To further investigate the identity of these proliferative cells, a double immunofluorescence staining was performed. Double staining of CD31 and PCNA showed a higher density of PCNA^+^CD31^+^ capillaries in the ChGel^MSC−Exo^ group than the control and ChGel^PBS^ groups on both day 7 and day 21 (white arrows, Fig. 6D, E). A similar result of dual staining with α-SMA was also seen in the PCNA^+^ cells. A higher density of the α-SMA^+^PCNA^+^ vascular smooth muscle cells in the ChGel^MSC−Exo^ group was demonstrated on both day 7 and day 21 (white arrows, Fig. 6F, G). Taken together, these results demonstrate that the population of CD31^+^ endothelial/endothelial progenitor cells and α-SMA^+^ vascular smooth muscle cells consist of the main part of the PCNA^+^ cells within the submucosa, indicating the enhancement of tissue and capillaries regeneration ability after ChGel^MSC−Exo^ treatment. To confirm the angiogenesis activity of MSC-Exo, growth-arrested human microvascular endothelial cells (HMVECs) were seeded in a Matrigel-coated plate and treated with PBS, MSC-Exo (100 μg/mL), or VEGF (20 ng/mL) for 8 h in vitro. The mesh counts, the number of junctions and branches, and total length of branches per view were significantly higher in the MSC-Exo group than in the PBS group (Fig. 6H).

### ChGel^MSC−Exo^ treatment induced the M2b macrophage polarization at the submucosal layer of esophagus

**Fig. 7:**
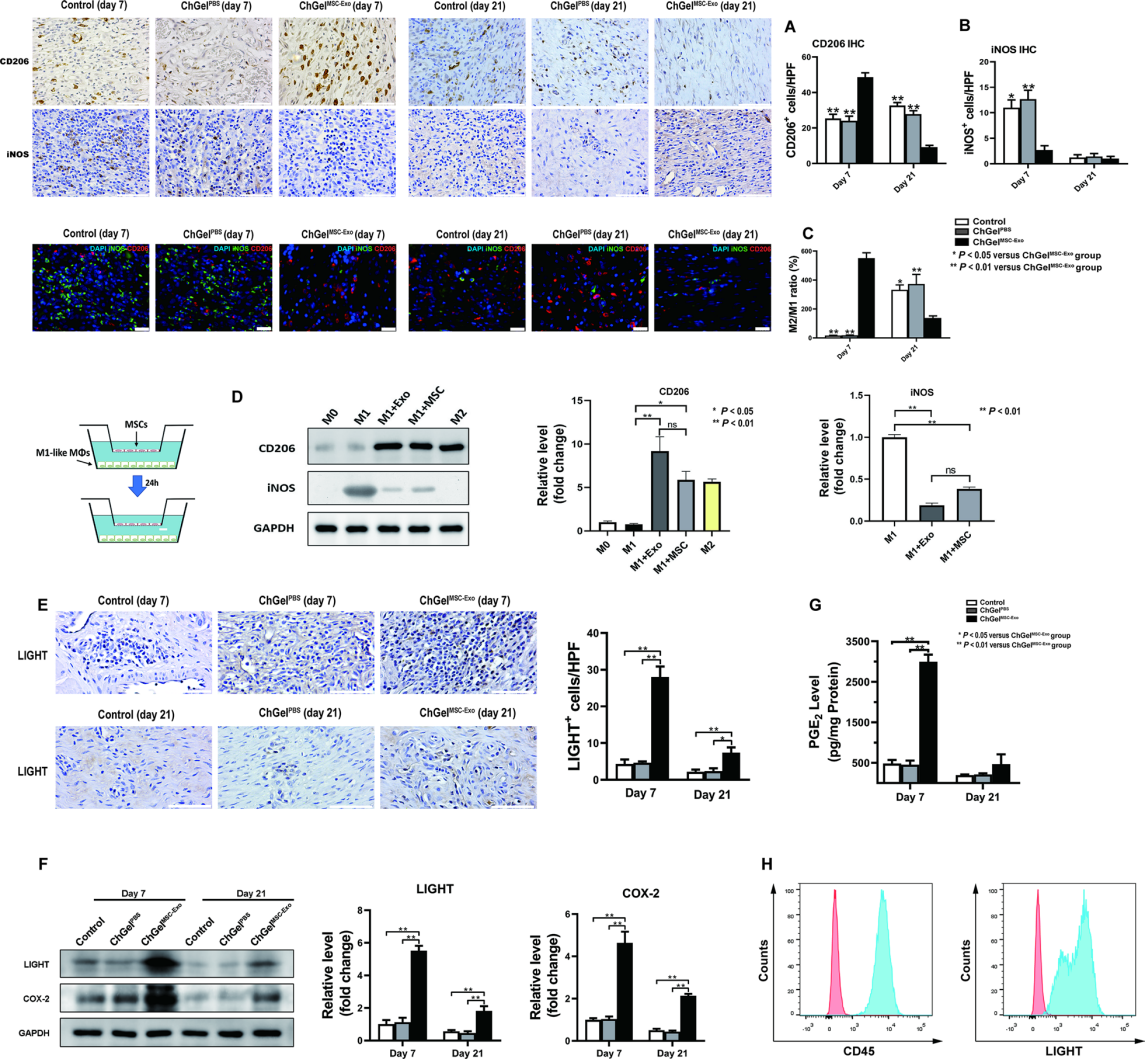
MSC-Exo treatment induces the M2b macrophage polarization at the submucosal layer of esophagus. **A**, **B** The quantification of the IHC on the expression of CD206 and iNOS on days 7 and 21 respectively. Scale bar, 100 μm. **C** Double immunofluorescence staining of iNOS and CD206 on day 7 and 21. Comparison of M2/M1-like MΦs ratios was conducted among the control, ChGel^PBS^ and ChGel^MSC−Exo^ groups on days 7 and 21. Scale bar, 25 μm. **D** Expression of M2 marker (CD206) and M1 marker (iNOS) in MSC/MSC-Exo treated M1-MΦs were examined by Western Blot. **E** The quantification of the IHC on LIGHT on days 7 and 21, scale bars, 100 μm. **F** The quantification of the Western blot on LIGHT, COX-2 on days 7 and 21. **G** ELISA analysis of PGE_2_ activity of wound samples obtained on days 7 and 21 respectively. **H** Flow cytometry showed the macrophage general marker CD45 and upregulation of M2b-specific marker LIGHT in MSC-Exo treated BMDMs in vitro. All data are shown as the means ± SEM. Statistical significance was analyzed by independent samples *t* test or one-way ANOVA followed by a Tukey post hoc analysis between two or multiple groups, respectively. *P < 0.05, **P < 0.01

Activation and phenotype polarization of macrophages (MΦs) play key roles in the initiation and resolution of fibrosis [26]. To understand the immunomodulatory effects of ChGel^MSC−Exo^ treatment, M1 and M2-like MΦs transitions in esophageal samples were investigated. ESD operation led to an obvious aggregation of MΦs at the ulcer margin area. The CD206^+^ M2-like MΦs mostly appeared upon ChGel^MSC−Exo^ treatment on day 7 compared with the control and ChGel^PBS^ groups, while on day 21 these cells were barely identified in the ChGel^MSC−Exo^ group (Fig. 7A). Meanwhile, the density of iNOS^+^ M1-like MΦs in the ChGel^MSC−Exo^ group was significantly smaller than in the control and ChGel^PBS^ groups on day 7, while on day 21 there was no significance among the three groups (Fig. 7B). Double immunofluorescence staining of iNOS and CD206 showed the consistent results; ChGel^MSC−Exo^ treatment induced an earlier and significant dominance of the immunomodulatory M2-like MΦs on day 7 (Fig. 7C), while the control and ChGel^PBS^ groups showed a delayed aggregation of M2-like MΦs which occurred on day 21 (Fig. 7C).

Studies in vitro were then performed to verify these findings. BMDMs and Raw 264.7 were remarkably positive for macrophage-specific surface markers CD68 and F4/80 (Additional file 1: Fig. S5A). MSC-Exo labelled with PKH26 was added into the culture medium of Raw 264.7. After 12 h, fluorescent microscopy showed the intracellular red fluorescence signals in Raw 264.7, suggesting the process of the cellular uptake of exosomes by macrophages (Additional file 1: Fig. S5B). Meanwhile, MSC-Exo at different concentrations (0/mL, 1 × 10^9^/mL, 2.5 × 10^9^/mL, 5 × 10^9^/mL, 1 × 10^10^/mL) was added into the culture medium of BMDMs respectively. Fluorescence microscopy detected the CD206 expression after treatment for 48 h, suggesting the polarization of macrophages towards M2 phenotype upon exosomes treatment in vitro (Additional file 1: Fig. S5C). And this effect was through a dosage-dependent manner (Additional file 1: Fig. S5C). Western blot and PCR analysis further confirmed that the protein and transcriptional levels of CD206 were also up-regulated with the increase in the amount of exosomes treatment respectively (Additional file 1: Fig. S5D, E). We next explored whether MSC-Exo could promote the polarization from M1 to M2 phenotype in macrophages. Cells were firstly stimulated by IFN-γ (40 ng/mL) and LPS (100 ng/mL) for 48 h to induce the M1 polarization, followed by treatment with MSC-Exo (1 × 10^10^/mL) or co-cultured with MSCs through the transwell culture inserts for 24 h. Immunofluorescence analysis revealed that the signal of M2 marker CD206 was significantly higher after co-culturing with MSCs or MSC-Exo (Additional file 1: Fig. S5F). Flow cytometry also detected a higher percentage of CD206^+^ BMDMs after treatment with MSCs or MSC-Exo (Additional file 1: Fig. S5G). Similarly, Western blot revealed that co-culturing with MSCs or exosomes significantly increased the expression of CD206 protein while attenuated the iNOS expression in the M1-like macrophages (Fig. 7D). These data together suggest that treatment with MSC-Exo significantly promoted the macrophages towards the anti-inflammatory M2-like polarization both in vivo and in vitro.

In fact, M2 macrophages have been further identified with three subsets, namely M2a, M2b, M2c [27, 28]. All subsets are considered to exert different roles in tissue repair and anti-inflammatory ability through secreting cytokines and chemokines, but can be distinguished by their biological function. M2a/c subclass could be beneficial in early wound healing stages but deleterious during the late phase of tissue remodeling responsible for fibrosis formation due to the over-expression of TGFβ1. In contrast, the M2b macrophages potentially exert both anti-inflammatory and anti-fibrotic effects through the release of COX_2_ [29–31]. In this study, histology and western blot analysis were further conducted to identify the expression of LIGHT protein (marker for M2b macrophage) on the esophageal samples. Significant up-regulation of LIGHT was demonstrated in the ChGel^MSC−Exo^ group compared with the control and ChGel^PBS^ group respectively (Fig. 7E, F). Increased expression of both COX_2_ and PGE_2_ was found after ChGel^MSC−Exo^ treatment on day 7, while there was no significant difference on PGE_2_ level among the three groups on day 21(Fig. 7F, G). Flow cytometry further demonstrated that BMDMs were remarkably positive to CD45 as well as to the M2b-specific marker LIGHT after treatment with MSC-Exo (1 × 10^10^/mL) for 48 h (Fig. 7H). Collectively, these results imply that co-culturing with MSC-Exo was able to promote the macrophages towards M2 polarization, especially the M2b subset, with a mitigated secretion of the profibrotic effector TGFβ1.

**Fig. 8:**
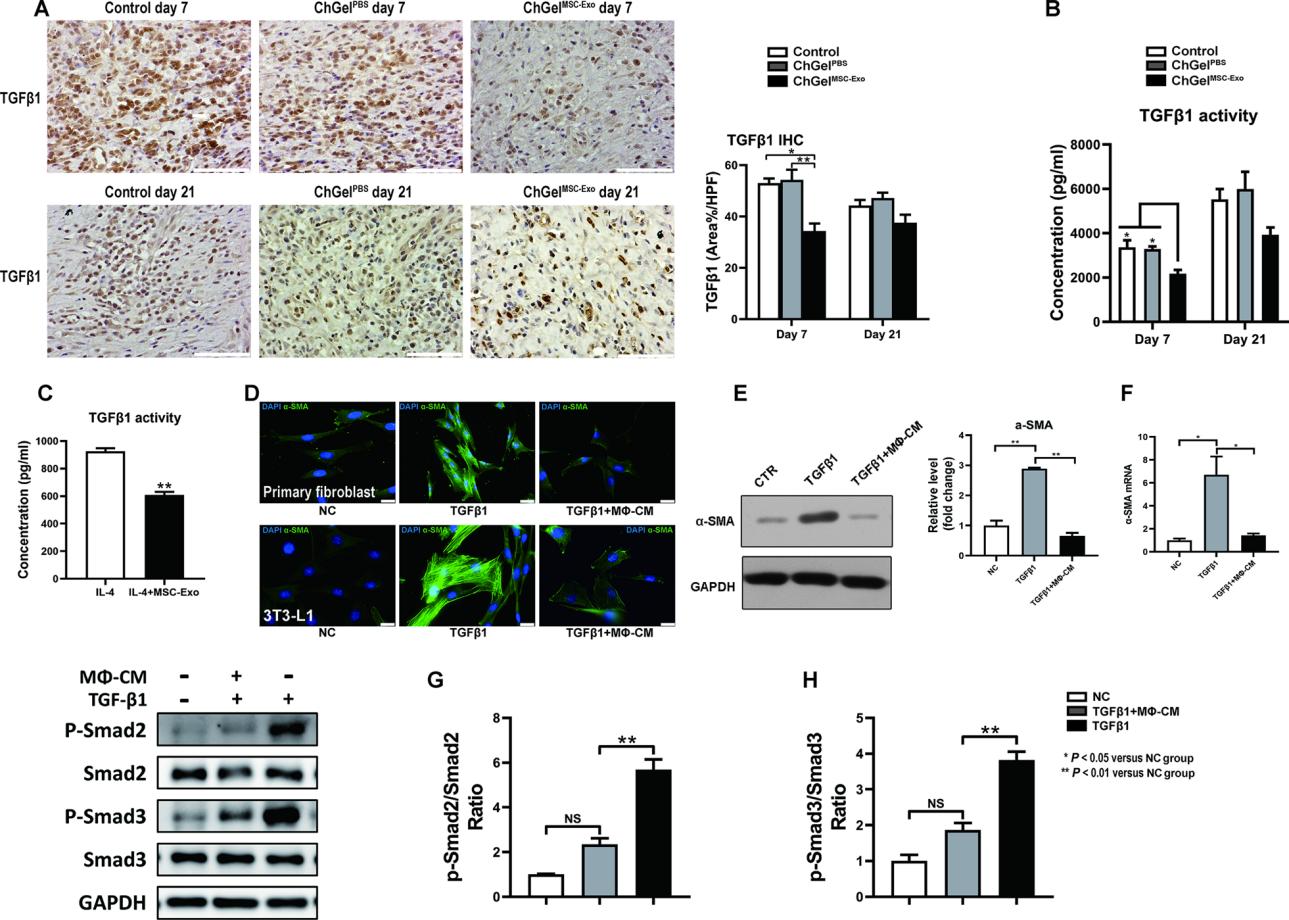
M2b macrophage polarization alleviates the fibroblast-myofibroblasts transition responsible for collagen accumulation. **A** The quantification of the IHC on TGFβ1 on day 7 and 21 respectively, scale bars, 100 μm. **B** ELISA analysis of TGFβ1 activity of wound samples obtained on day 7 and 21 respectively. **C** Co-administration of MSC-Exo (1 × 10^10^/mL) led to a significant reduction of TGFβ1 levels in the IL-4-induced M2-BMDMs in vitro. **D**–**F** Effect of MΦ-CM on fibrolast transition. The expression of α-SMA in fibroblasts was determined by immunofluorescence (Scale bars, 25 μm), Western blot and real-time quantitative PCR, respectively. **G**, **H** Western blot analysis of the effect of MΦ-CM on TGFβ1/Smad2/Smad3 signaling in fibroblast cells. All data are shown as the means ± SEM. Statistical significance was analyzed by one-way ANOVA followed by a Tukey post hoc analysis. *P < 0.05, **P < 0.01

It is in line with our findings in vivo, which showed a significantly inhibited protein level of TGFβ1 in the ChGel^MSC−Exo^ group compared with the control and ChGel^PBS^ groups on day 7 (*vs* control, *P* < 0.05 and *vs* ChGel^PBS^ group, *P* < 0.01 for IHC respectively; Fig. 8A) (*vs* control, *P* < 0.05 and *vs* ChGel^PBS^ group, *P* < 0.05 for ELISA respectively; Fig. 8B). For experiments in vitro, M2-BMDMs were firstly induced by IL-4 (20 ng/mL) treatment. ELISA assay then displayed that these M2-BMDMs exhibited notably elevated TGFβ1 activity. Intriguingly, co-administration of MSC-Exo (1 × 10^10^/mL) led to a significant reduction of TGFβ1 levels by 40% in the IL-4-induced M2-BMDMs (Fig. 8C), suggesting that while MSC-Exo may promote M2-like macrophage polarization, it concurrently reduces the expression of TGFβ1.

### The M2b macrophage polarization alleviated the fibroblast-myofibroblasts transition responsible for the collagen accumulation

The mechanism of TGFβ1 promoting fibrosis may be associated with the pro-differentiation of the resident fibroblasts to myofibroblasts, and probably relies on the activation of TGFβ1/Smad2/Smad3 pathway [32]. In this study, we further determined the possible effects of the M2b MΦs-derived conditioned medium (MΦ-CM) on the fibroblast-myofibroblast transition. Treatment of the primary fibroblasts with TGFβ1 (5 ng/mL) for 3 days significantly promoted the expression of α-SMA, the specific marker for myofibroblast (Additional file 1: Fig. S6A–C). However, co-culturing with MΦ-CM [final concentration of 10% (v/v)] significantly alleviated the TGFβ1-induced α-SMA expression at both the protein (Fig. 8D, E) and transcriptional levels (Fig. 8F). In addition, TGFβ1 treatment led to a significant increase in the p-Smad2 and p-Smad3 levels, while co-culturing cells with MΦ-CM significantly reduced the activation of TGFβ1/Smad2/Smad3 signaling (Fig. 8G, H). Taken together, these results imply that MSC-Exo treatment significantly induce the polarization of the M2b macrophages, subsequently suppressing the fibroblast-myofibroblast transition and inhibiting the collagen production and fibrotic progression.

### Exosomal MFGE8 activated the STAT3 pathway to induce the M2 macrophage polarization

Exosomes can be efficiently taken in by the recipient cells, thereby regulating cell activities by transporting biomolecules, including protein, DNA, mRNAs, and microRNA [33]. In this study, liquid chromatography-tandem mass spectrometry (LC–MS/MS) was conducted to elucidate the protein profiles of ADMSC-Exo from pigs. A total of 425 proteins were detected in the profiling. Asporin (ASPN) was set as the control since the intensity value was at the median among all the identified proteins. The functions of these proteins were predicted by performing Gene Ontology (GO) and Kyoto Encyclopedia of Genes and Genomes (KEGG) analysis. Results showed that the exosomal proteins were mainly enriched in the regulation of single-organism process in biological process (BP), extracellular region in cell component (CC), and protein binding in molecular function (MF) (Additional file 1: Fig. S7A). The KEGG pathway investigation suggested that these proteins primarily enriched in the proteasome, phagosome and complement and coagulation cascades processes (Additional file 1: Fig. S7B). Cytokines and chemokines with an intensity value higher than twofold of Asporin were then considered as the high expression. Results showed that MFGE8, which was considered a factor involved in M2 macrophage polarization, was the most abundant effector among all the exosomal proteins (Additional file 1: Fig. S7C). Previous studies indicated that MFGE8 could activate the STAT-3 pathway and characterize the M2 polarization of macrophages [34, 35]. To understand the possible mechanism of MSC-Exo in inducing M2 MΦs polarization, BMDMs were treated with MSC-Exo (1 × 10^10^/mL), with the presence or absence of MFGE8 blocking antibody and STAT3 inhibitor S3I-201. Results showed that p-STAT3 were significantly upregulated after treatment with MSC-Exo (Additional file 1: Fig. S7D), whereas p-STAT3 levels were significantly reduced upon co-culturing with anti-MFGE8 or S3I-201(Additional file 1: Fig. S7D), indicating that the exosomal MFGE8 may play an important role in promoting M2 macrophage polarization through activation of the STAT3 pathway.

### Pig-ADMSC-Exo miRNA profiling and their putative functions by bioinformatics analysis

**Fig. 9:**
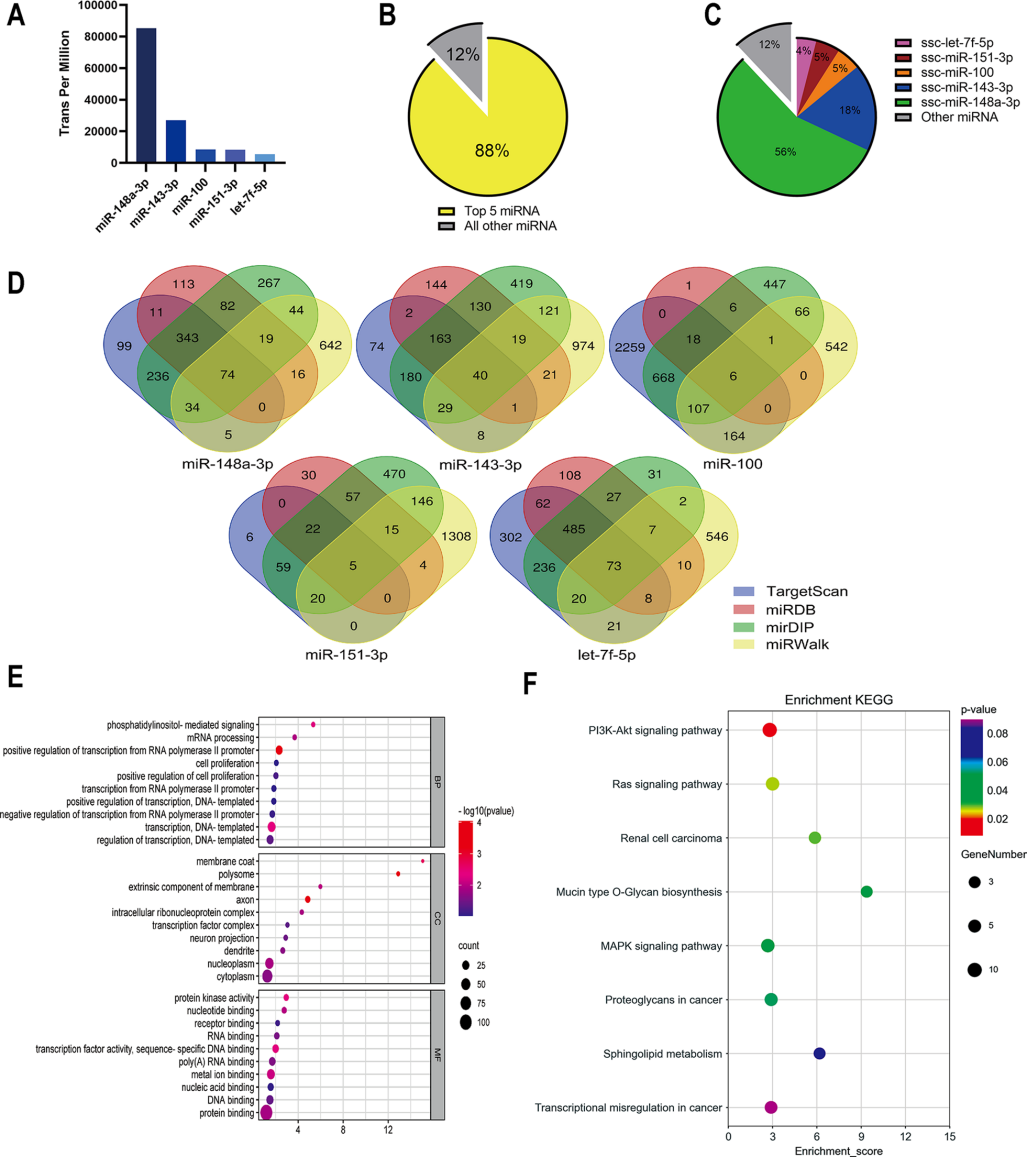
pig-ADMSC-Exo miRNA profiling and bioinformatics analysis. **A** The five top detected pig-ADMSC-Exo miRNAs were illustrated by histogram. **B** The percentage of the five top detected miRNAs in total miRNAs. **C** The proportion of each of the five miRNAs in total miRNAs. **D** Venn diagram showed the predicted target genes of the five most abundant miRNAs. **E** GO analysis showing the 30 most enriched GO terms for these target genes. **F** KEGG pathway analysis showing the 8 most enriched pathways for these target genes

It is reported that exosomes exert the therapeutic effects mainly by delivering miRNAs [36]. To explore the potential effective miRNAs of the pig-ADMSC-Exo for the prevention of esophageal stricture, we profiled and quantified the miRNAs content of pig-ADMSC-Exo by high-throughput miRNA sequencing. As shown in Fig. 9A, the top five abundant miRNAs (ssc-miR-148a-3p, miR-143-3p, ssc-miR-100, ssc-miR-151-3p and ssc-let-7f-5p) were identified in pig-ADMSC-Exo based on the transcripts per million (TPM). These top five miRNAs occupied about 88% of the total miRNAs (Fig. 9B). Among them, ssc-miR-148a-3p, miR-143-3p, ssc-miR-100, ssc-miR-151-3p and ssc-let-7f-5p accounted for 56%, 18%, 5%, 5%, 4% of the total miRNAs respectively (Fig. 9C). To further explore the function of these most abundant miRNAs, we performed GO and KEGG enrichment analysis. The target genes were predicted and the intersection was illustrated in the Venn diagram. A total of 198 genes were obtained as the predicted genes targeted for the most abundant miRNAs (Fig. 9D). GO analysis demonstrated that these predicted target genes were mainly enriched in the processes of positive regulation of transcription from RNA polymerase II promoter in biological process (BP), protein binding in molecular function (MF), and cytoplasm in cellular component (CC), respectively (Fig. 9E). The KEGG evaluation demonstrated that these target genes were primarily enriched in the PI3K (phosphatidylinositol 3 kinase) -Akt signaling pathway (Fig. 9F). PI3K-Akt signaling pathway is well known to regulate several aspects of metabolic functions that were crucial for the wound healing, including cell survival, growth, migration, and protein synthesis [37]. In conclusion, these data indicate that pig-ADMSC-Exo may alleviate esophageal stricture through delivering miRNA into resident cells, thus regulating the PI3K-Akt signaling pathway.

### Exosomal miR‑148a‑3p promotes angiogenesis and proliferation in endothelial cells

Since miR-148a-3p was detected as the most abundant miRNA in pig-ADMSC-Exo, further studies were conducted to determine its role in promoting angiogenesis and cellular proliferation, by which such biological processes are crucial for mucosal healing [38]. Vascular endothelial cell line HMVECs were transfected with miR-148a-3p mimic, mimic control, miR-148a-3p inhibitor and inhibitor control respectively. The expression of miR-148a-3p was detected by RT-PCR (Additional file 1: Fig. S8A). MTS assay showed that up-regulation of miR-148a-3p significantly promoted cellular proliferation by 48 h while down-regulation of miR-148a-3p inhibited such effect (*P* < 0.01 and *P* < 0.01, respectively; Additional file 1: Fig. S8B). Tube formation assay indicated that HMVECs transfected with miR-148a-3p mimics displayed significantly increased tubule formation and branch length, whereas suppression of miR-148a-3p expression significantly hindered the angiogenic capability of the cells (*P* < 0.01 and *P* < 0.05, respectively; Additional file 1: Fig. S8C).

## Discussion

Incorporation of exosomes into material scaffolds for target delivery has been considered a possible approach on the development of biocompatible medical implants. One advantage is the prolonged retention of exosomes in performing their function in the wound areas [39]. In this study, our results showed that MSC-Exo delivered by ChGel sponge scaffold mitigated esophageal stricture through enhancing angiogenesis and reducing fibrosis and inflammation after near-circumferential ESD; ChGel^MSC−Exo^ group showed an average mucosa stricture rate reducing to around 30% of that in the control or ChGel^PBS^ group on day 21, suggesting its considerable prospect in the management of patients who may develop severe esophageal stricture due to the extensive mucosal defect after ESD, especially in those who are contraindicatory or intolerable towards the current available therapeutic approaches.

Balloon dilations, temporary stent and steroids are commonly used for management of esophageal stricture after ESD, but their effectiveness would be limited by the need of repeated endoscopic dilatation, potential risks of perforation and stent migration [6, 7]. Other novel treatment methods were examined recently [40, 41]. Tomonori et al. reported a biodegradable esophageal stent for stricture prevention, while the long-term efficacy by 24 weeks was unsatisfactory due to the stricture recurrence as well as the complications such as esophago-bronchial fistula [42]. Tissue engineering approaches including the autologous oral cell sheet transplantation, thigh skin-grafting, and cell sheet transplantation were also developed [43–45]. The incidence of stricture was reported to be 44% in patients received autologous esophageal mucosa treatment after circumferential ESD [44], but this technology is relatively expensive and labor-intensive. Autologous skin or esophageal mucosa grafting is an aggressive approach with relatively low patient compliance; recurrent esophageal stricture was found in 8 out of 9 patients (88.9%) during the average follow-up period of 24.7 days. [45] Takeshi et al. recently reported a gel consisted of the carboxymethyl cellulose (5%) and the conditioned medium from MSCs (MSC-CM), which after oral administration could accelerate the healing of mucosal defect after semi-circumferential ESD, thereby relieving the esophageal stricture by approximately 24–28% in porcine models [20]. However, such delivery approach may present challenges in ensuring comprehensive coverage of the entire mucosal defect circumference, as well as in accurately assessing the extent of drug retention at the wound site, thereby potentially compromising the efficacy of therapy. In the current study, we designed a porous sponge scaffold as an effective means to endoscopically deliver the MSC-derived exosomes to the wound site after near-circumferential ESD. Experiments in vitro and in vivo have confirmed that exosomes could be gradual released from the sponge scaffold and significantly taken in by tissue cells on 12 h, thereby ensuring the efficacy of MSC-derived exosomes in preventing the development of esophageal stricture.

TGFβ1 is the cytokine which triggers the differentiation of resident fibroblasts to myofibroblasts, hence playing an important role in developing fibrosis [46]. Myofibroblast is the key cell type responsible for collagen production during extracellular matrix remodeling [47, 48]. The pro-inflammatory factors such as IL-1β activate the profibrotic macrophages to produce TGFβ1 during tissue injury [26]. Modulating the paracrine profile of the macrophages might be a promising approach in alleviating fibrosis development [49]. The current study showed that ChGel^MSC−Exo^ treatment induced a M2 biased polarization of the resident macrophages at the submucosa of esophagus (Fig. 7). Early transition from the proinflammatory M1-like MΦs to the immunomodulatory M2-like MΦs has been recognized as a crucial event in wound healing [50], and a delayed transition might cause fibrosis and scarring reactions and inhibit tissue regeneration [39, 50]. Here, double immunofluorescence analysis (CD206 and iNOS) demonstrated that the ChGel^MSC−Exo^ treatment induced an earlier and significant transition of immunomodulatory M2-like MΦs on day 7 compared with the control and ChGel^PBS^ groups. Despite the leukocyte infiltration score was observed to be comparable among the three groups on day 7, ChGel^MSC−Exo^ treatment significantly inhibited the expression of IL-1β while concurrently increased the IL-10 level. Previous studies demonstrated that M2 macrophage promoted fibroblast-to-myofibroblast activation by secreting the pro-fibrotic TGF-β1 and PDGF, resulting in increased α-SMA and collagen I expression in myofibroblasts [26]. Intriguingly, our results showed that the TGF-β1 activity in the wound samples obtained on day 7 was significantly lower in the ChGel^MSC−Exo^ group than the control and ChGel^PBS^ groups. In vitro ELISA analysis also demonstrated that TGFβ1 activity was reduced in the culture supernatant of M2-like MΦs after treatment with MSC-Exo. These results imply that ChGel^MSC−Exo^ treatment might be able to promote polarization of the M2-like MΦs with a mitigated secretion of TGFβ1. Upon further examination of the subsets of M2 macrophages (M2a, M2b, and M2c [27–29]) induced by MSC-Exo treatment, our findings revealed a notable increase in the expression of LIGHT (M2b marker), COX_2_ and PEG_2_ subsequent to the ChGel^MSC−Exo^ treatment on both days 7 and 21 when compared with the control and ChGel^PBS^ groups. These results suggest that ChGel^MSC−Exo^ treatment may tend to promote the polarization of the M2b-MΦ subset specifically, which is associated with both anti-inflammatory and anti-fibrotic properties. [29, 30]. Our data was consistent with previous literature, showing that MSC-Exo treatment attenuated colon mucosal inflammation and fibrosis by polarizing M2b-MФs [51].

Exosome contains an assortment of components including proteins, DNA, mRNA, and microRNA. Due to the phospholipid membrane structure, exosomes can be efficiently taken in by the recipient cells, thereby regulating cell activities by transporting biomolecules [33, 52]. Several exosomal miRNAs including miR-182, miR-223 and miR-let7 were identified with the ability to promote M2 macrophage polarization via activating signal transducer and activator of transcription-3 (STAT3), STAT6 and peroxisome proliferator-activated receptor-gamma (PPARγ) pathways [52]. Cytokines derived from MSC-Exo such as IL-10, PGE_2_ and insulin-like growth factor 2 (IGF-2) were also reported to promote M2 polarization [52, 53]. In this study, LC/MS analysis found that the MFGE8 was the most abundant factor in the exosomes released from MSCs. MFGE8 is a glycoprotein expressed in nearly all cell types and is known to facilitate phagocytic clearance of apoptotic or dead cells [35]. Recent studies reported that MFGE8 could enact anti-inflammatory effects by promoting M2 macrophages polarization [34, 35]. Integrin β3 is a known receptor of MFGE8, and the activation of integrin β3/SOCS3/STAT3 signaling pathway has been shown to induce M2 macrophages polarization [35]. In the current study, our results demonstrated that exosomal MFGE8 significantly induced STAT-3 phosphorylation in macrophages and further promoted M2b polarization. These findings are in line with the previous studies, which demonstrated MFGE8 mediated reprogramming of macrophages toward M2 polarization by increasing phosphorylation of STAT-3 [34, 35]. In addition, we investigated other exosomal components that were crucial for wound healing. miRNA sequencing showed that ssc-miR-148a-3p accounted for 56% of the total miRNAs in pig-ADMSC-Exo. Wang et al. [54] reported that miR-148a-3p significantly promoted tissue angiogenesis through activation of the EGFR/MAPK signaling pathway, which is consistent with our KEGG enrichment analysis. In addition, KEGG analysis with the target genes of the top 5 miRNAs in pig-ADMSC-Exo revealed that PI3K-Akt pathway was most enriched among the top 8 enriched pathways. PI3K-Akt signaling pathway assumes significance due to its pivotal involvement in cellular functions such as survival, proliferation, and migration, all of which are critical for mucosal healing [37].

This study has several limitations. Firstly, the optimal dosing and course of treatment remain to be optimized. Pigs in the current study received the two rounds of endoscopic intervention with ChGel^MSC−Exo^ immediately after ESD and on day 7 consecutively. Nevertheless, our results indicate a noteworthy impact of exosomes therapy in mitigating esophageal stricture, particularly evident during the early stage (day 7), which might suggest that a single administration of MSC-Exo at the dosage of 1 mg could effectively initiate tissue repair and reduce fibrotic progression after esophageal ESD. Secondly, studies are necessary to validate the efficacy of exosomes derived from non-human primates and human sources in mitigating esophageal stricture after ESD, since variations in the cargo contents may arise from MSCs of diverse species.

In summary, our study demonstrated that endoscopic delivery of the sponge scaffold encapsulated with ADMSC-Exos prevented esophageal stricture after near-circumferential ESD through alleviating collagen production, suppressing inflammation and promoting angiogenesis. Exosomes treatment significantly induced the early polarization of the anti-inflammatory M2-like MΦs especially the M2b subset, which attenuated the fibroblast-myofibroblast transition, while exosomal MFGE8 was identified to be crucial in modulating this process. Our findings suggest that endoscopic delivery of the MSC-Exo-encapsulated sponge scaffold may serves as a promise for preventing esophageal stricture after ESD, especially in patients with low compliance or contraindications to the conventional therapeutic approaches such as ballon dilation and steroid administration.

## Methods

### Study design

Male or female domestic pigs with a body weight of 25 to 30 kg were used in this study (pigs, *n* = 24). This study was based on a randomized controlled design and multivariate analysis was used to minimize potential confounders. One ulcer was created in each pig and follow-up endoscopic surveillance was conducted every week to evaluate stricture level. The sample size was estimated by reduction of mucosal constriction rate based on a previous study [20] (effect size: 30%, α = 0.05, and power = 0.80). In-vitro experiments were conducted to explore key signaling pathways involved in mediating the main cellular events responsible for the fibrosis process. All in vitro experiments were repeated at least three times. Blind method is used for histopathological evaluation and analysis of immunohistochemistry (IHC) and immunofluorescence images.

### Isolation and characterization of ADMSCs and MSCs-derived exosome

Under general anesthesia, subcutaneous fat tissues were taken from the pigs. Tissues were then washed three times with sterile phosphate buffered solution (PBS), followed by treating with 0.1% type I collagenase in PBS for 30 min at 37 ℃ with gentle agitation. After filtration through a 100 µm mesh filter to remove debris, the filtrate was centrifuged at 300×*g* for 5 min. The pellet was then washed three times with PBS and completely suspended in Dulbecco’s modified Eagle’s medium (DMEM) (Low glucose; Gibco) supplemented with 10% fetal bovine serum (FBS, Gibco), antibiotics (100 U/mL penicillin, 100 µg/mL streptomycin, and 0.25 μg/mL Amphotericin B; Gibco), and 2 mM l-glutamine. The cultures were maintained in an incubator with a humidified atmosphere of 5% CO_2_. The cell phenotyping of the MSCs were tested with anti-CD29, CD90, CD105, CD44 and CD45. The adipogenic, chondrogenic and osteogenic differentiation potentials of ADMSC in vitro were tested.

MSCs were cultured in the medium with the exosome depleted FBS (Gibco; 10%) when the cell confluence reached about 80–90%. The culture supernatant was then harvested after 48 h. The extraction of the MSCs-derived exosomes was conducted using differential centrifugation based on previous protocol [55]. Briefly, the supernatants were centrifuged at 2000*g* for 10 min and then 10000*g* for 30 min to remove the debris and apoptotic bodies. Subsequently, the supernatants were centrifuged at 100,000*g* for 70 min, followed by washing with PBS and purification by centrifugation at 100,000*g* for 70 min again. All the centrifugations were performed at 4 ℃. The pellet was suspended in PBS and sterilized by filtration through a 0.22 μm filter. The concentration of total exosome was qualified using Bradford assay.

Exosomes were characterized by western blot to detect the specific protein markers (Alix, CD63, Tsg101 and HSC70). The distribution of particles sizes and quantity was inferred from the nanoparticle tracking analysis (NTA) using the NanoSight system (Malvern NS500, UK). In addition, the morphology of exosomes was further observed under the transmission electron microscopy (TEM). Briefly, the purified exosomes resuspended in PBS were fixed with 2% paraformaldehyde for half an hour at room temperature. 10 μL samples were then added to a copper grid and dyed with 1% uranyl acetate (UA) for 5 min at room temperature. Afterwards, the dried grid was examined using a Hitachi H-7700 (Hitachi, Japan) electron microscope at 120 kV. For exosome quality control and to reduce batch-to-batch variability, MSC-Exo that was validated to inhibit fibroblast-to-myofibroblast transformation in vitro was applied in each experiment. 500 μg of exosomes in 100 μL PBS were slowly and evenly dropped into the lyophilized ChGel sponge (5 × 5 × 5 mm), and then placed in a tube containing 1 mL PBS at 37 °C. The controlled release of encapsulated MSC-Exo was investigated by immersing sponge with PBS for 0.5 h, 2 h, 3 h, 6 h, 8 h, 12 h, 15 h. The released exosomes were detected in PBS buffer using BCA protein quantitative assay.

### Preparation and characterization of chitosan/gelatin sponges

Chitosan/gelatin (ChGel) sponges were prepared by lyophilized technique. A total of 2.5 g chitosan (Aladdin, China) was dissolved in a beaker containing 100 mL distilled water, followed by adding 1 mL absolute acetic acid and homogenization at 1000 rpm for 30 min to form a transparent solution. Then, 1 g gelatin (Aladdin, China) was slowly added to the solution with vortexing (1000 rpm) for 15 min. The final solution was transferred inside a cylindrical mold with a diameter of 2.5 cm and length of 10 cm without liquid bubbles. The scaffold was then frozen at – 80 ℃ overnight, followed by lyophilization for 48 h to obtain the ChGel sponges. The sponges were neutralized with 1 M NaOH, washed with distilled water and lyophilized again before use.

The surface of the sponges was observed through scanning electron microscopy (SEM; Hitachi SU8010, Japan). Briefly, 50 μg of exosomes in 10 μL PBS were slowly and evenly dropped into a 2 mm^3^ lyophilized ChGel sponge. The scaffold was then frozen at – 80 ℃ overnight, followed by lyophilization for 48 h to obtain the Exos-coated ChGel sponges. To assess the degradability of the sponges, samples were incubated in HCL solution (pH 3.0) at 37 ℃ and the time when the sponge was completely dissolved in the solution was recorded. The absorbability of sponge (swelling rate) was detected by weighing the sponge after immersing into the PBS at different times.

### Development of esophageal stricture after ESD in porcine models

Pigs (20–30 kg, *n* = 24) were fasted for 18 h before endoscopy. ESD procedures were performed by an endoscopist with experience on more than 1000 cases. Under general anesthesia, esophageal ESD was performed using a single channel GI endoscope (GIF-HQ190; Olympus, Tokyo, Japan). An artificial lesion with 4/5 circumference and 10 cm in length in the lower esophagus was marked using a Dual-knife (Olympus) and the electrosurgery unit. The lesion was then elevated with submucosal injection with saline. Afterwards, the marked area was safely cut from the distal and proximal side to the lateral side of the lesion, followed by the dissection of the submucosal layer. Slight bleeding was controlled using hemostatic forceps (Olympus).

### Functional evaluations of ChGel sponges on esophageal stricture post-ESD

After developing the esophageal lesion, animals were randomly assigned into three groups. For the MSC-Exo treatment group (ChGel^MSC−Exo^ group, *n* = 8), endoscopic delivery of ChGel sponges was performed to shield the esophageal wound and fixed by deploying endoscopic clips on the proximation. Then 1 mg ADMSCs-derived exosomes resuspended in 10 mL PBS were slowly injected into the sponge by the endoscopic injector. For the ChGel^PBS^ group (*n* = 8), pigs were treated with the ChGel sponges encapsulating 10 mL PBS solution. There were the other 8 pigs without any treatment to serve as the controls (control group, *n* = 8). Each group was divided into two subgroups based on the date of sacrifice: d 7 (*n* = 3 each) and d 21 (*n* = 5 each). The sponges were expected to keep attachment onto the esophageal wound for 8–12 h. After the procedures, pigs were given analgesic and parenteral nutrition (197 kcal/kg/day) for 48 h. Animals were then allowed to liquid diet on day 3, gradually transiting to the normal diet from day 4. Endoscopic surveillance was performed every week to monitor the ulcer healing process and evaluate the esophageal stricture. The body weight of pig was also recorded.

### Sample collection

Biopsy were conducted on days 7 and 14 to draw wound samples for western blot and PCR assays. On day 7, pigs (*n* = 3/group) were euthanized to obtain full-thickness esophageal samples for histological analysis. The other pigs (*n* = 5/group) were sacrificed on day 21. The resected esophagus was immediately placed on a corkboard and fixed with pins. The degree of stricture at the wound area was expressed as mucosal constriction rate expressed by the following formula [56]:

$$\mathrm{Mucosal\, constriction\, rate }\,(\mathrm{\%}) = \left\{1-\frac{\mathrm{length\, of\, short\, axis\, at\, site\, of\, maximal\, constriction}}{\left(\mathrm{length\, of\, short\, axis\, at\, a\, normal\, mucosal\, site\, on\, upper\, side}+\mathrm{length\, of\, short\, axis\, at\, a\, normal\, mucosal\, site\, on\, a\, lower\, side}\right)\times \frac{1}{2}}\right\}\times 100$$

The measurement of stricture diameter was performed blindly and averaged by 3 measurers independently. Samples of ulcer margin were harvested through endoscopic biopsy. Afterwards, specimens were fixed in 10% formalin for 24 h for histological evaluation. For western blot, PCR, or ELISA evaluation, specimens were immediately snap-frozen in liquid nitrogen and transferred to – 80 ℃.

### In vitro biocompatibility test

50μL composites containing 200 μg chitosan and 100 μg gelatin was added to the culture medium (2 mL), followed by seeding the Raw 264.7 or 3T3-L1 cells (ATCC) at a density of 1 × 10^5^ cells/mL. After culturing for 48 h, cells were stained with calcein AM and PI (Thermo Scientific) for 15 min at 37 ℃, washed with PBS and captured using a fluorescence microscope (Olympus IX83, Japan).

### Isolation of mouse bone marrow-derived macrophages (BMDMs)

BMDMs were isolated and differentiated as previously described [57]. Briefly, C57B/L mice were euthanized by overdose isoflurane. The hind limbs were disinfected with 75% ethanol for 3 min and then isolated the limbs bones with a sterile scissor, followed by washing with sterile PBS in the culture hood. The bone marrow cavity was rinsed with cold PBS using a 28-G needle for several times until it turned to be semi-translucent. The flushing fluid was collected and filtered through a 40 μm strainer, followed by centrifugation at 300*g* for 5 min at room temperature. Subsequently, the pellet was suspended in DMEM, and seeded on 6-well plates with a concentration of 10^6^ cells per well. The harvested cells were then cultured in the complete DMEM containing M-CSF (20 ng/mL) for 5–7 days to stimulate cell differentiation from monocytes to macrophages. Cells were stained with the marker F4/80 and CD68 to assess the purity before further analysis.

### The regulation of macrophage polarization

To induce the M1 and M2 activation, RAW 264.7 cells or BMDMs were cultured with IFN-γ (40 ng/mL) and LPS (100 ng/mL), as well as IL-4 (20 ng/mL), for 24 h respectively. Afterwards, the medium was changed, and cells were cultured in the serum-free DMEM for another 24 h. Such macrophage cells were considered as polarized cells, and the culture medium was collected and prepared as the polarized conditioned medium for the following experiments.

### Preparation of the M2b macrophage-derived condition medium [MΦ-CM]

M2b-macrophages were firstly induced through MSC-Exo treatment (1 × 10^10^/mL for 48 h). Flow cytometry was conducted to confirm the expression of LIGHT, the M2b-MΦ specific marker. Macrophage-derived condition medium was then prepared as previously described [58]. In brief, when M2b macrophage cells reached around 80% confluence, the culture medium was changed to the DMEM supplemented with 0.5% FBS. After 48 h, the culture supernatant was collected and centrifuged at 3000 rpm for 15 min. The MΦ-CM was then filtered by a 0.22 μm filter (Sigma) for sterilization.

### Western blot

Samples were lysed using CytoBuster™ Protein Extraction Reagent (Calbiochem). Protein extracts were heat-denatured at 100 ℃ for 10 min, separated through sodium dodecyl sulfate–polyacrylamide gel electrophoresis (SDS-PAGE) and transferred to PVDF membranes (Sigma). After blocking with 5% BSA for 1 h at room temperature, the membranes were incubated with primary antibody against GAPDH (CST; 1:1000), Albumin (CST; 1:1000), TSG101 (Abcam; 1:500), CD63 (Abcam; 1:500), HSC70 (Santa Cruz; 1:500), Calnexin (Invitrogen; 1:500), α-SMA (Abcam; 1:3000), collagen I (Abcam; 1:1000), Smad2 (CST; 1:1000), p-Smad2 (CST; 1:1000), Smad3 (CST; 1:1000), p-Smad3 (Abcam; 1:1000), CD206 (Abcam; 1:1000), Arg1 (CST; 1:1000), iNOS (Abcam; 1:1000), LIGHT (Santa Cruz; 1:500), COX-2 (CST; 1:500), STAT3 (Abcam; 1:1000) and p-STAT3 (Abcam; 1:1000) at 4 ℃ overnight, followed by the secondary antibodies for 1 h at room temperature. Protein bands were detected by chemiluminescence using ChemiDoc (Bio-Rad), and the density was analyzed using Image J software. Each experiment was repeated 3 times.

### Quantitative real-time PCR

Samples were lysed with TRIzol (Life Technologies). Extraction of RNA and reverse transcription of cDNA (EvoScript Universal cDNA Master, Roche) were conducted according to the manufacturer’s instructions. Real-time qPCR was performed on a QuantStudio 7 Real-Time PCR System (Applied Biosystems) with SYBR green PCR Master Mix (Invitrogen). Results were calculated using the 2^−ΔΔCt^ method. The sequences of primers were listed as follow (5′ → 3′): CD206, forward *CCATCTCAGTTCAGACGGCA*, reverse *CATACAGGGTGACGGAAGCC*; α-SMA (mouse), forward *AGAGGCACCACTGAACCCTA*, reverse *AGAGTCCAGCACAATACCAGT*; VEGF, forward *GACGAAGGTCTGGAGTGTGT*, reverse *CAAGGCCCACAGGGATTTTCT*; IL-10, forward *AAAAGGGGGACAACAGTAGGTG*, reverse *GGCTGGTTGGGAAGTGGATG*; IL-1β1, forward *CCACAAATCTCTAGTGCTGGC*, reverse *AGGGTGGGCGTGTTATCTTT*; COL1A2, forward *AGGGCATTAGGGGTCACAAC*, reverse *ACAGGACCCACACTTCCATC*; COL3A1, forward *GGCAGGGAACAACTGATGGT*, reverse *GACTGACCAAGATGGGAGCA*; α-SMA (pig), forward *CCCTCCTGAGCGCAAATACT*, reverse *GGCTTCGTCGTACTCCTGTT;* LIGHT (pig), forward *AGAAGCTGATACAAGAGCGGAG*, reverse *TAATAGTAGCCGGCCTTGGT*.

### Immunohistochemistry (IHC) and immunofluorescence

Sections were deparaffinized, and the antigen retrieval was performed for 20 min at 98 °C in citrate buffer (10 mM, pH = 6.0). Sections were then blocked in 2.5% Normal Goat Serum Blocking Solution (Vector) for 1 h at room temperature, followed by incubation with primary antibodies (α-SMA, 1:500; Col-1, 1:400; Fibronectin, 1:200; CD31, 1:200; PCNA, 1:200; Arg1, 1:200; CD206, 1:900; iNOS, 1:50; TGFβ1, 1:100; F4/80, 1:200; LIGHT, 1:200) overnight at 4℃. For IHC, the biotinylated secondary antibody (Vector) was applied, and the signal detection was performed using diaminobenzidine (DAB) solution (Vector). For immunofluorescence, sections were incubated with FITC-conjugated secondary antibody (Invitrogen) for 1 h at room temperature. Cell nuclei were counterstained with 4′,6-diamidino-2-phenylindole (DAPI; Invitrogen).

### Hematoxylin and eosin (H&E), Mason’s trichrome and Sirius Red staining

Esophagus tissue sections were dewaxed and stained with hematoxylin & eosin (H&E) staining to evaluate the histological features of inflammation. Leukocyte infiltration was graded according to the previous literature: grade 1, mild focal leukocytic infiltration; grade 2, dense but focal leukocytic infiltration; grade 3, dense and diffuse leukocytic infiltration involving more than 50% of ulcer base [14]. Esophagus tissue sections were stained with Mason's trichrome and Sirius Red staining to evaluate the accumulation of collagen fibers, followed by polarized light mode of microscope to selectively highlight collagen networks [59]. The detailed procedures were performed according to the manufacturer’s protocols (Masson’s trichrome staining kit, Abcam; Direct Red 80 Dye, Sigma).

### Tube formation assay

Human microvascular endothelial cells (HMVECs; ATCC) were starved (1% FBS) for 24 h. The growth factor-reduced Matrigel (Corning) was thawed and coated on a 24-well plate (250 μL/well). HMVECs were then seeded in each well at a density of 7 × 10^4^ per well followed by treatment with PBS, MSC-Exo (100 μg/mL), or VEGF (20 ng/mL; Santa Cruz) for 8 h. Afterwards, pictures for each well were captured and the Image J software was used for analysis. All experiments were carried out three times.

### ELISA assay

To determine the activity change of TGFβ1 and PGE2 in esophagus tissues after exosomes treatment, ELISA assay was applied. Tissues were homogenized in assay buffer and centrifuged to collect supernatant at 16,000×*g* for 15 min. Assays were then performed using ELISA kits (R&D Systems) according to the manufactures’ instructions.

### Statistical analysis

Results were displayed as means ± SEM or median (interquartile range) accordingly. P-P plot and Shapiro–Wilk test were used to evaluate the normality of distributions. Independent samples *t* test was conducted to compare the data between two groups. One-way analysis of variance (ANOVA) was performed, followed by Tukey multiple comparisons post hoc test to determine the statistical significance among multiple groups. Changes in the body weight was examined by repeated-measures analysis of variance (repeated ANOVA), and a post hoc multivariate test was performed at each time point. Kruskal–Wallis test (non-parametric ANOVA) followed by Dunn’s multiple comparison post hoc test was used to analyze the leukocyte infiltration scoring. SPSS (Statistical Package for the Social Sciences) 20.0 was applied for statistical analysis, and *P* < 0.05 on both sides was considered statistically significant. All authors had access to the study data and reviewed and approved the final manuscript.

### Supplementary Information

**Additional file 1: Figure S1.** The characterization of ADMSCs and the detection of ADMSCs differentiation. **A** Morphology of ADMSCs at passage 0. **B** Flow cytometry for CD markers including CD29, CD44, CD90, CD105 and negative marker CD45. **C** Differentiation in vitro for adipogenesis, osteogenesis, and chondrogenesis. Scale bars, 50 μm. **Figure S2.** Scheme of fabricating the ChGel sponge scaffold. **Figure S3.** Biocompatibility of ChGel sponge scaffold. **A**, **B** Comparison of cell viability between PBS group and ChGel group through live/dead staining assay in RAW264.7 and 3T3-L1 cells respectively (scale bars, 50 μm). **Figure S4** Treatment with ChGel^MSC-Exo^ alleviates fibrosis, inflammatory and promotes angiogenesis at the submucosal layer of esophagus. **A**–**F** The quantification of the western blot on α-SMA, collagen-I, p-Smad2, IL-10, IL-1β and VEGF on day 7, 14 and 21. All data are shown as the means ± SEM. Statistical significance was analyzed by one-way ANOVA followed by a Tukey *post hoc* analysis. *P < 0.05, **P < 0.01. **Figure S5.** Effect of MSC-Exo on macrophage phenotype polarization. **A** Morphology of BMDMs and RAW 264.7; Flow cytometry was conducted to detect the macrophage markers (F4/80 and CD68) in BMDMs and RAW264.7 respectively. **B** Red fluorescence signals in Raw264.7 showed the process of cellular uptake of PKH26-labelled MSC-Exo. **C**–**E** The ability of MSC-Exo on promoting M2-like macrophage polarization was through a dosage-dependent manner, as tested by immunofluoresence, Western blot and PCR analysis respectively. **F**, **G** Immunofluorescence and flow cytometry were conducted to analyze the expression of CD206 on the previously polarized M1 macrophages subsequent to exposure to MSC/MSC-Exo. All data are shown as the means ± SEM. Statistical significance was analyzed by one-way ANOVA followed by a Tukey *post hoc* analysis. *P < 0.05, **P < 0.01. Scale bars, 50 μm. **Figure S6.** Concentration titration and time effect of TGFβ1 on fibroblast-myofibroblast transition. **A**, **B** Analysis of the fibroblasts subject to the treatment with the varying concentration of TGFβ1 by immunofluorescence and western blot. **C** Western blot analysis of the TGFβ1 stimulation on fibroblasts across different time points. All data are shown as the means ± SEM. Statistical significance was analyzed by one-way ANOVA followed by a Tukey *post hoc* analysis among multiple groups. *P < 0.05, **P < 0.01. Scale bar, 25 μm. **Figure S7.** Exosomal MFGE8 activates the STAT3 pathway and induces the M2 macrophage polarization. **A** Go (Gene Ontology) analysis of LC-MS/MS results was conducted to elucidate the protein functions of ADMSC-Exo. **B** The top 10 most enriched pathways were identified by KEGG pathway analysis. **C** Representative intensity value of the exosomal proteins with relatively high expression, which exceeded a 2-fold increase in comparison to Asporin. **D** Protein levels of p-STAT3 and STAT3 in the control (BMDMs without any treatments), MSC-Exo treatment (BMDMs cultivated with MSC-Exo), MSC-Exo+Anti-MFGE8 (BMDMs cultivated with MSC-Exo and Anti-MFGE8) and MSC-Exo+S3I-201 (BMDMs cultivated with MSC-Exo and S3I-201) groups; Comparison of p-STAT3/STAT3 ratio was conducted among these four groups. All data are shown as the means ± SEM. Statistical significance was analyzed by one-way ANOVA followed by a Tukey *post hoc* analysis among multiple groups. *P < 0.05, **P < 0.01. **Figure S8.** miR-148a-3p promotes proliferation and angiogenesis in HMVECs. **A** Expression of miR-148a-3p was detected by qPCR in HMVECs transfected with miR-148a-3p mimic or inhibitor. **B** MTS assay to test proliferation ability of HMVECs transfected with miR-148a-3p mimic or inhibitor. **C** The angiogenesis capacity of HMVECs transfected with miR-148a-3p mimic or inhibitor was evaluated by tube formation assay. All data are shown as the means ± SEM. Statistical significance was analyzed by independent samples *t* test or one-way ANOVA followed by a Tukey *post hoc* analysis between two or multiple groups, respectively. *P < 0.05, **P < 0.01. Scale bars, 100 μm. **Table S1.** Full gene names for pig cytokines and chemokines. **Table S2.** Full name of genes related to fibrosis.

**Additional file 2: Movie S1.** The ChGel scaffold encapsulated with MSC-Exo were delivered endoscopically to shield the esophageal wound immediately after ESD.

## Data Availability

The datasets used and/or analyzed during the current study are available from the corresponding author on reasonable request.

## Abbreviations

ABL1: C-abl oncogene 1, non-receptor tyrosine kinase
ACTA2: Actin, alpha 2, smooth muscle, aorta
AGT: Angiotensinogen-like
AKT1: V-akt murine thymoma viral oncogene homolog 1
BCL2: B-cell CLL/lymphoma 2
Bmp7: Bone morphogenetic protein 7
CAV1: Caveolin 1, caveolae protein, 22 kDa
CCL11: Chemokine (C–C motif) ligand 11
CCL2: Chemokine (C–C motif) ligand 2
CCL3L1: Chemokine (C–C motif) ligand 3-like 1
CTGF: Connective tissue growth factor
CCR2: Chemokine (C–C motif) receptor 2
CCR7: Chemokine (C–C motif) receptor 7
CEBPB: CCAAT/enhancer binding protein (C/EBP), beta
JUN: C-JUN protein
COL1A2: Collagen, type I, alpha 2
COL3A1: Collagen, type III, alpha 1
CREBBP: CREB binding protein
CXCR4: Chemokine (C–X–C motif) receptor 4
DCN: Decorin
EDN1: Endothelin 1
EGF: Epidermal growth factor
