# Supplementary material for: MFGE8 in exosomes derived from mesenchymal stem cells prevents esophageal stricture after endoscopic submucosal dissection in pigs
Source: J Nanobiotechnology. 2024 Apr 1;22:143. doi: 10.1186/s12951-024-02429-0 (PMC10986023; doi:10.1186/s12951-024-02429-0)
Supplement: Supplementary file 1 — Additional file 1: Figure S1. The characterization of ADMSCs and the detection of ADMSCs differentiation. A Morphology of ADMSCs at passage 0. B Flow cytometry for CD markers including CD29, CD44, CD90, CD105 and negative marker CD45. C Differentiation in vitro for adipogenesis, osteogenesis, and chondrogenesis. Scale bars, 50 μm. Figure S2. Scheme of fabricating the ChGel sponge scaffold. Figure S3. Biocompatibility of ChGel sponge scaffold. A, B Comparison of cell viability between PBS group and ChGel group through live/dead staining assay in RAW264.7 and 3T3-L1 cells respectively (scale bars, 50 μm). Figure S4 Treatment with ChGelMSC-Exo alleviates fibrosis, inflammatory and promotes angiogenesis at the submucosal layer of esophagus. A–F The quantification of the western blot on α-SMA, collagen-I, p-Smad2, IL-10, IL-1β and VEGF on day 7, 14 and 21. All data are shown as the means ± SEM. Statistical significance was analyzed by one-way ANOVA followed by a Tukey post hoc analysis. *P < 0.05, **P < 0.01. Figure S5. Effect of MSC-Exo on macrophage phenotype polarization. A Morphology of BMDMs and RAW 264.7; Flow cytometry was conducted to detect the macrophage markers (F4/80 and CD68) in BMDMs and RAW264.7 respectively. B Red fluorescence signals in Raw264.7 showed the process of cellular uptake of PKH26-labelled MSC-Exo. C–E The ability of MSC-Exo on promoting M2-like macrophage polarization was through a dosage-dependent manner, as tested by immunofluoresence, Western blot and PCR analysis respectively. F, G Immunofluorescence and flow cytometry were conducted to analyze the expression of CD206 on the previously polarized M1 macrophages subsequent to exposure to MSC/MSC-Exo. All data are shown as the means ± SEM. Statistical significance was analyzed by one-way ANOVA followed by a Tukey post hoc analysis. *P < 0.05, **P < 0.01. Scale bars, 50 μm. Figure S6. Concentration titration and time effect of TGFβ1 on fibroblast-myofibroblast transition. A, B Analysis of the [file 12951_2024_2429_MOESM1_ESM.docx]

**Additional file Legends of Figures and Tables**


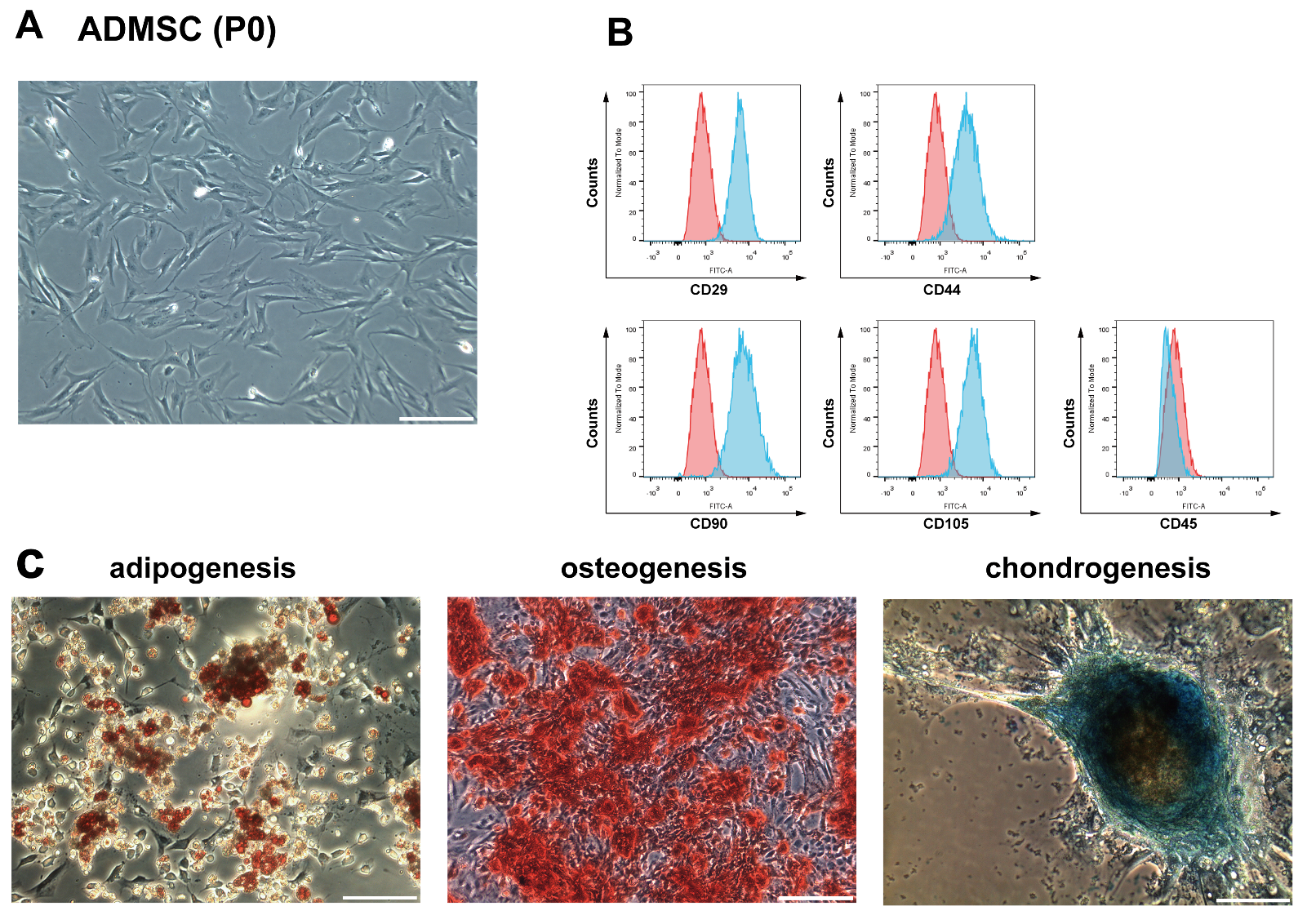


**Fig. S1.** **The characterization of ADMSCs and the detection of ADMSCs differentiation.** **(A)** Morphology of ADMSCs at passage 0. **(B)** Flow cytometry for CD markers including CD29, CD44, CD90, CD105 and negative marker CD45. **(C)** Differentiation in vitro for adipogenesis, osteogenesis, and chondrogenesis. Scale bars, 50μm.


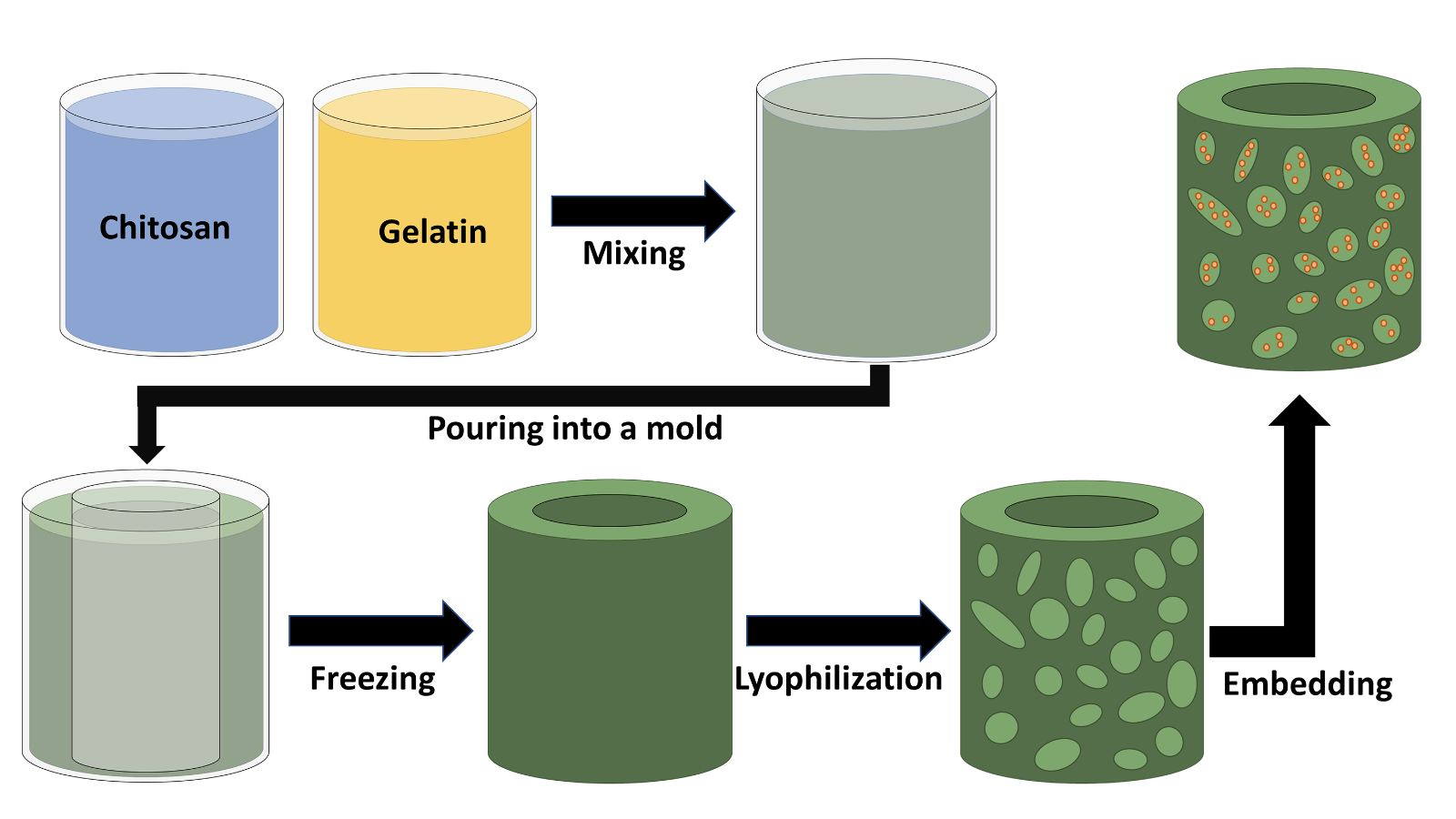


**Fig. S2.** **Scheme of fabricating the ChGel sponge scaffold.**


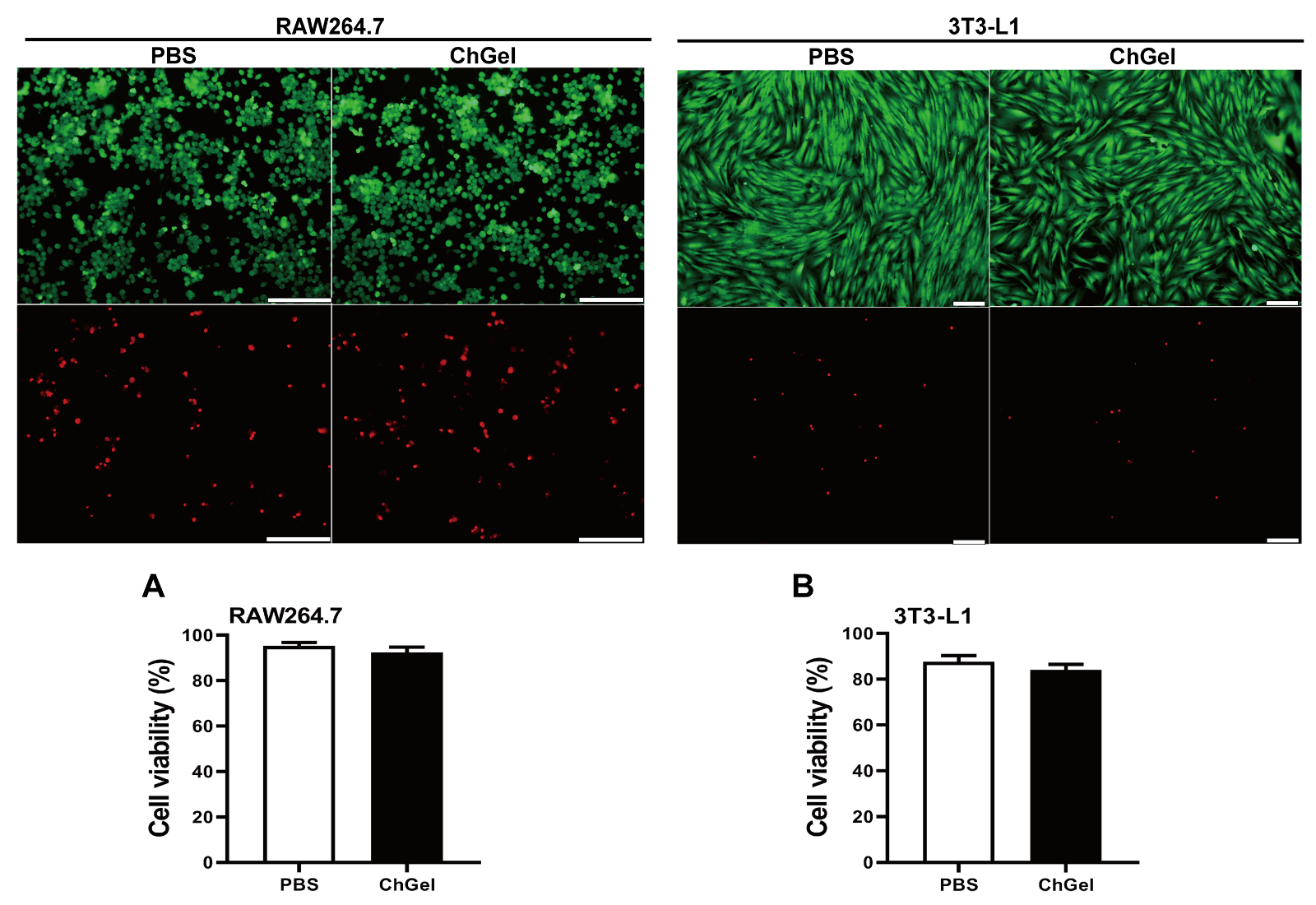


**Fig. S3.** **Biocompatibility of ChGel sponge scaffold. (A-B)** Comparison of cell viability between PBS group and ChGel group through live/dead staining assay in RAW264.7 and 3T3-L1 cells respectively (scale bars, 50μm).


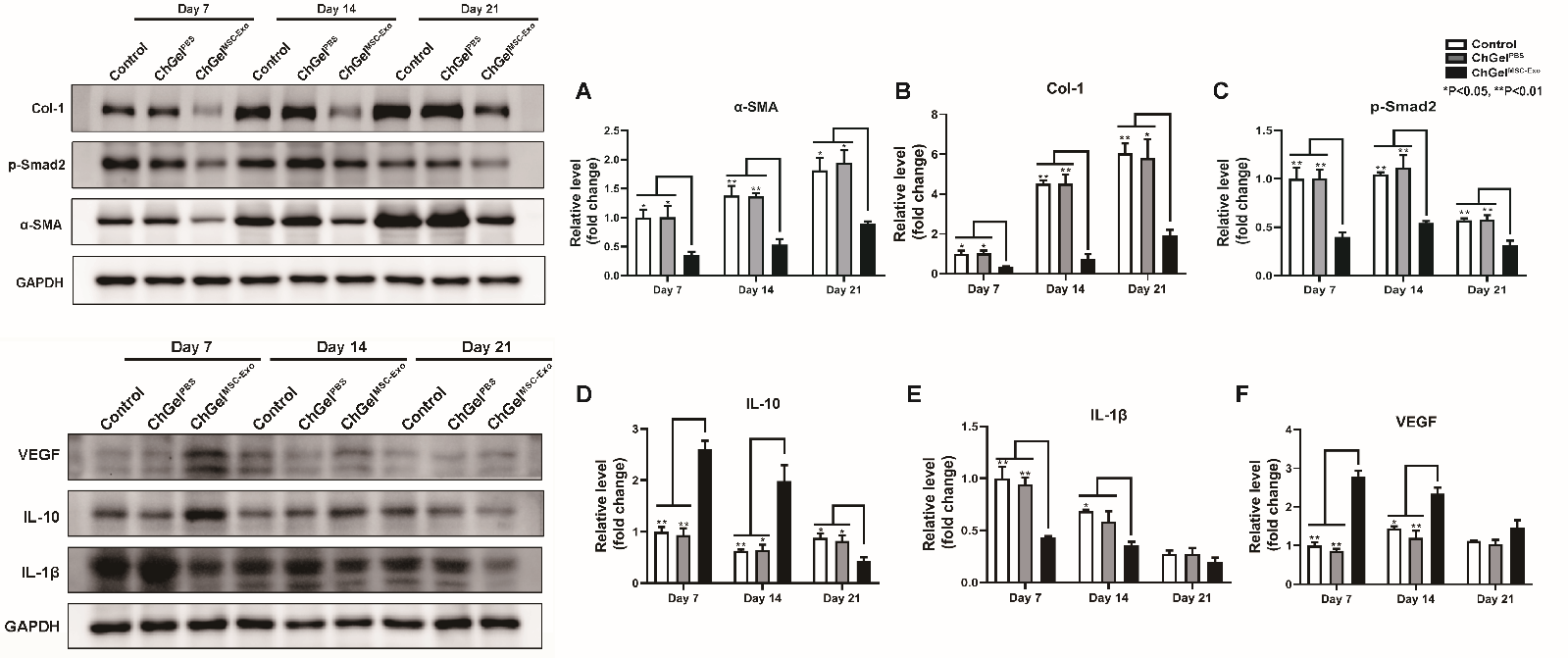


**Fig. S4. Treatment with ChGel^MSC-Exo^ alleviates fibrosis, inflammatory and promotes angiogenesis at the submucosal layer of esophagus.** **(A-F)** The quantification of the western blot on α-SMA, collagen-I, p-Smad2, IL-10, IL-1β and VEGF on day 7, 14 and 21. All data are shown as the means ± SEM. Statistical significance was analyzed by one-way ANOVA followed by a Tukey *post hoc* analysis. **P* < 0.05, ***P* < 0.01.


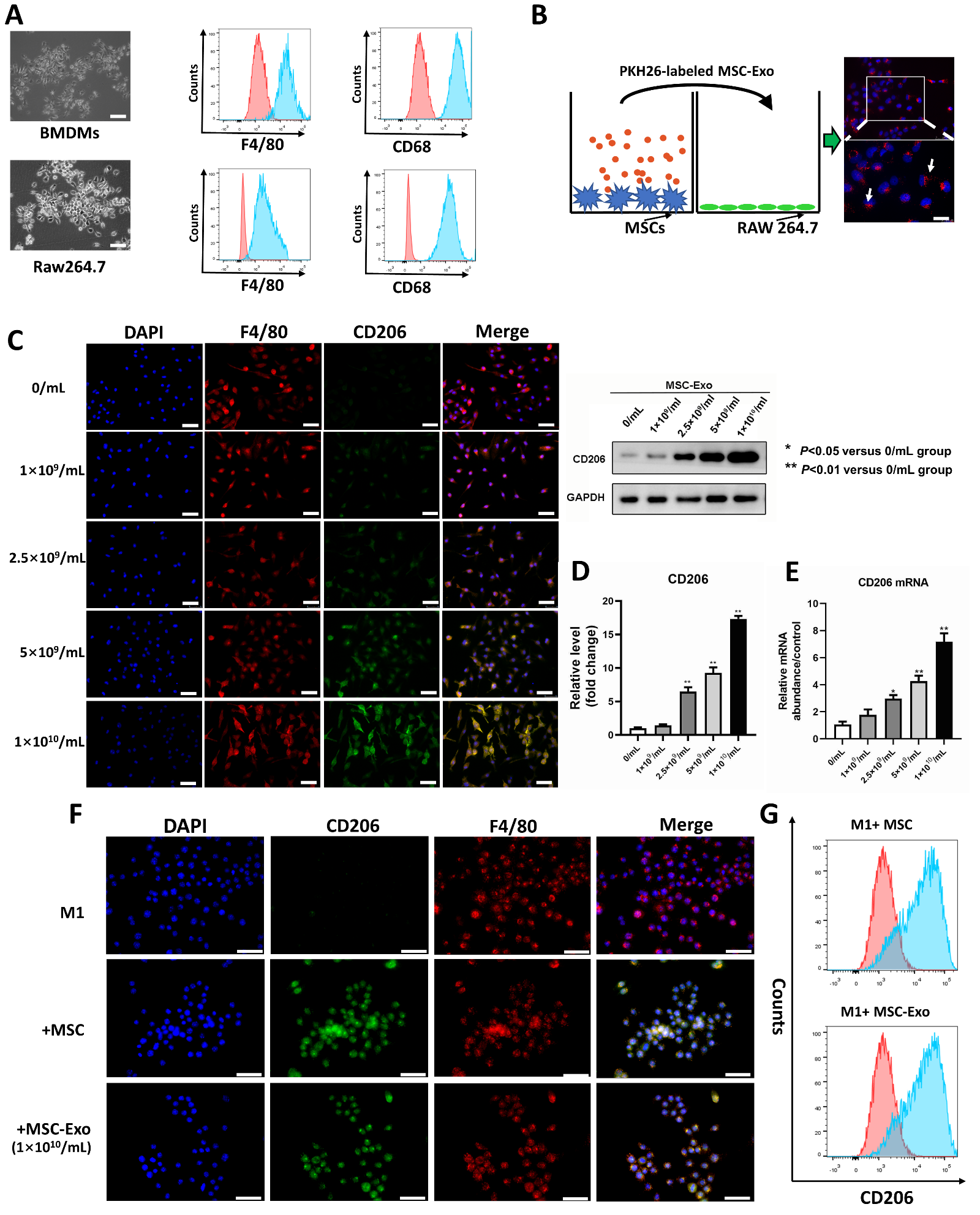


**Fig. S5.** **Effect of MSC-Exo on** **macrophage phenotype polarization. (A)** Morphology of BMDMs and RAW 264.7; Flow cytometry was conducted to detect the macrophage markers (F4/80 and CD68) in BMDMs and RAW264.7 respectively. **(B)** Red fluorescence signals in Raw264.7 showed the process of cellular uptake of PKH26-labelled MSC-Exo. **(C-E)** The ability of MSC-Exo on promoting M2-like macrophage polarization was through a dosage-dependent manner, as tested by immunofluoresence, Western blot and PCR analysis respectively. **(F-G)** Immunofluorescence and flow cytometry were conducted to analyze the expression of CD206 on the previously polarized M1 macrophages subsequent to exposure to MSC/MSC-Exo. All data are shown as the means ± SEM. Statistical significance was analyzed by one-way ANOVA followed by a Tukey *post hoc* analysis. **P* < 0.05, ***P* < 0.01. Scale bars, 50μm.


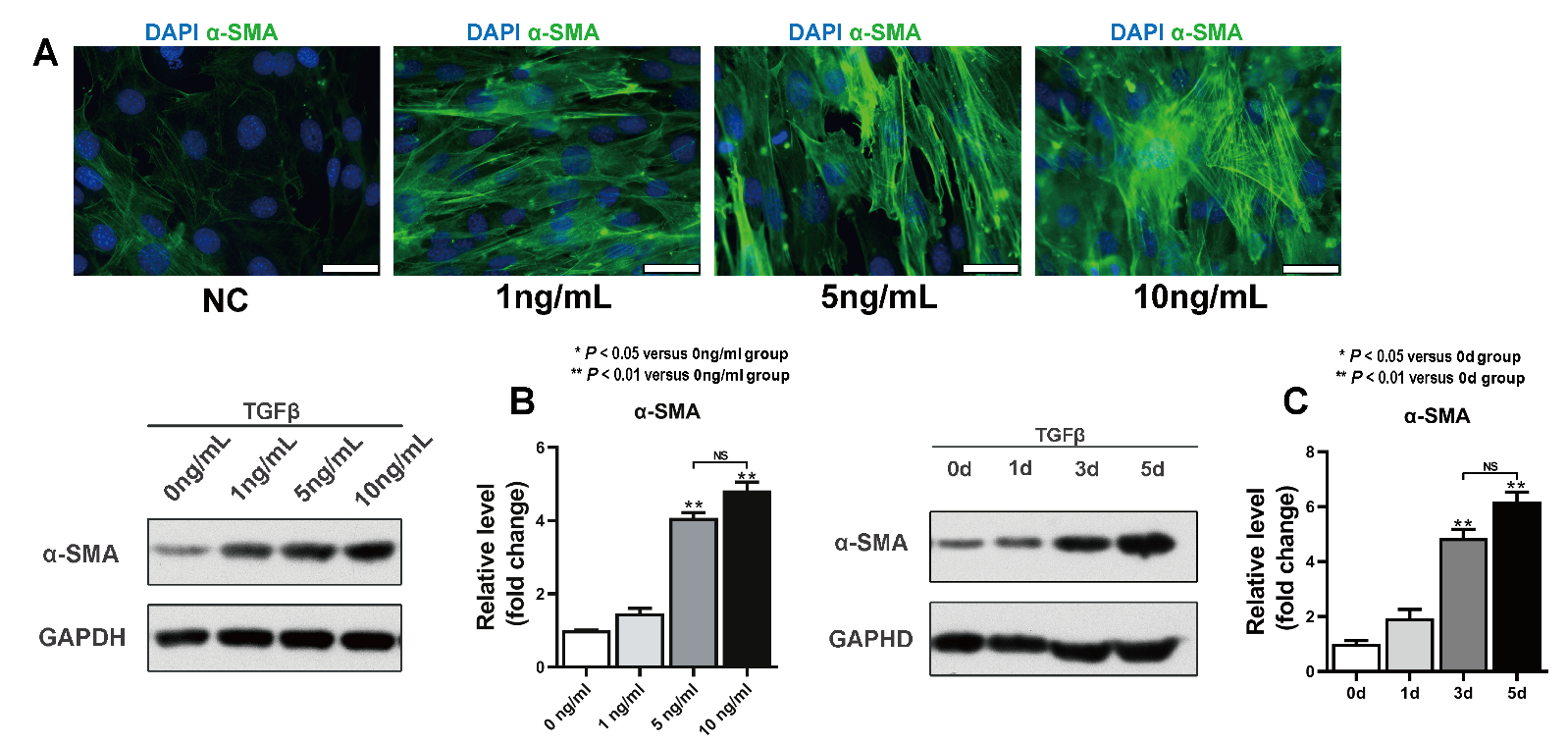


**Fig. S6.** **Concentration titration and time effect of TGFβ1 on fibroblast-myofibroblast transition.** **(A-B)** Analysis of the fibroblasts subject to the treatment with the varying concentration of TGFβ1 by immunofluorescence and western blot. **(C)** Western blot analysis of the TGFβ1 stimulation on fibroblasts across different time points. All data are shown as the means ± SEM. Statistical significance was analyzed by one-way ANOVA followed by a Tukey *post hoc* analysis among multiple groups. **P* < 0.05, ***P* < 0.01. Scale bar, 25μm.


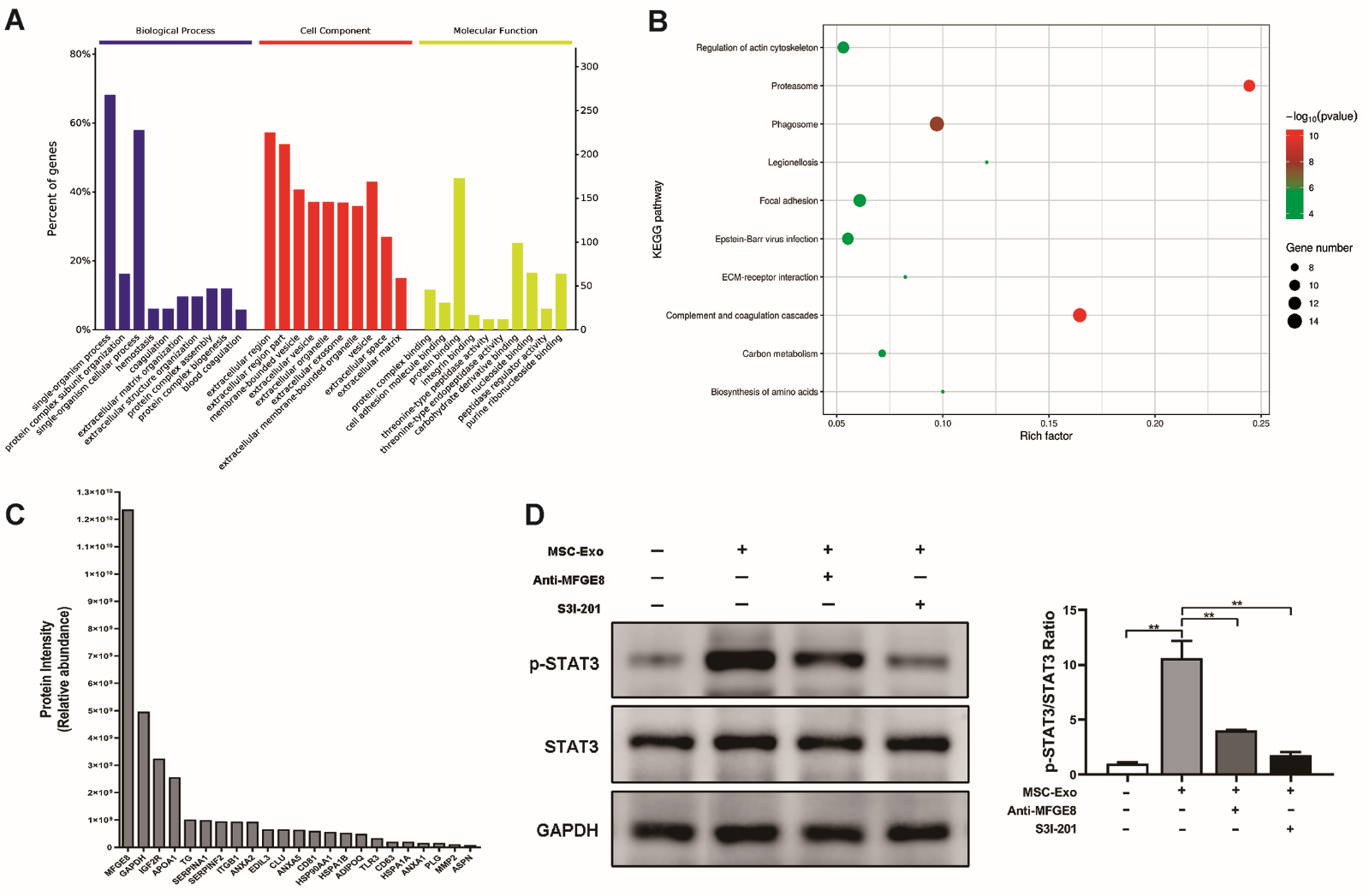


**Fig. S7. Exosomal MFGE8 activates the STAT3 pathway and induces the M2 macrophage polarization. (A)** Go (Gene Ontology) analysis of LC-MS/MS results was conducted to elucidate the protein functions of ADMSC-Exo. **(B)** The top 10 most enriched pathways were identified by KEGG pathway analysis. **(C)** Representative intensity value of the exosomal proteins with relatively high expression, which exceeded a 2-fold increase in comparison to Asporin. **(D)** Protein levels of p-STAT3 and STAT3 in the control (BMDMs without any treatments), MSC-Exo treatment (BMDMs cultivated with MSC-Exo), MSC-Exo+Anti-MFGE8 (BMDMs cultivated with MSC-Exo and Anti-MFGE8) and MSC-Exo+S3I-201 (BMDMs cultivated with MSC-Exo and S3I-201) groups; Comparison of p-STAT3/STAT3 ratio was conducted among these four groups. All data are shown as the means ± SEM. Statistical significance was analyzed by one-way ANOVA followed by a Tukey *post hoc* analysis among multiple groups. **P* < 0.05, ***P* < 0.01.


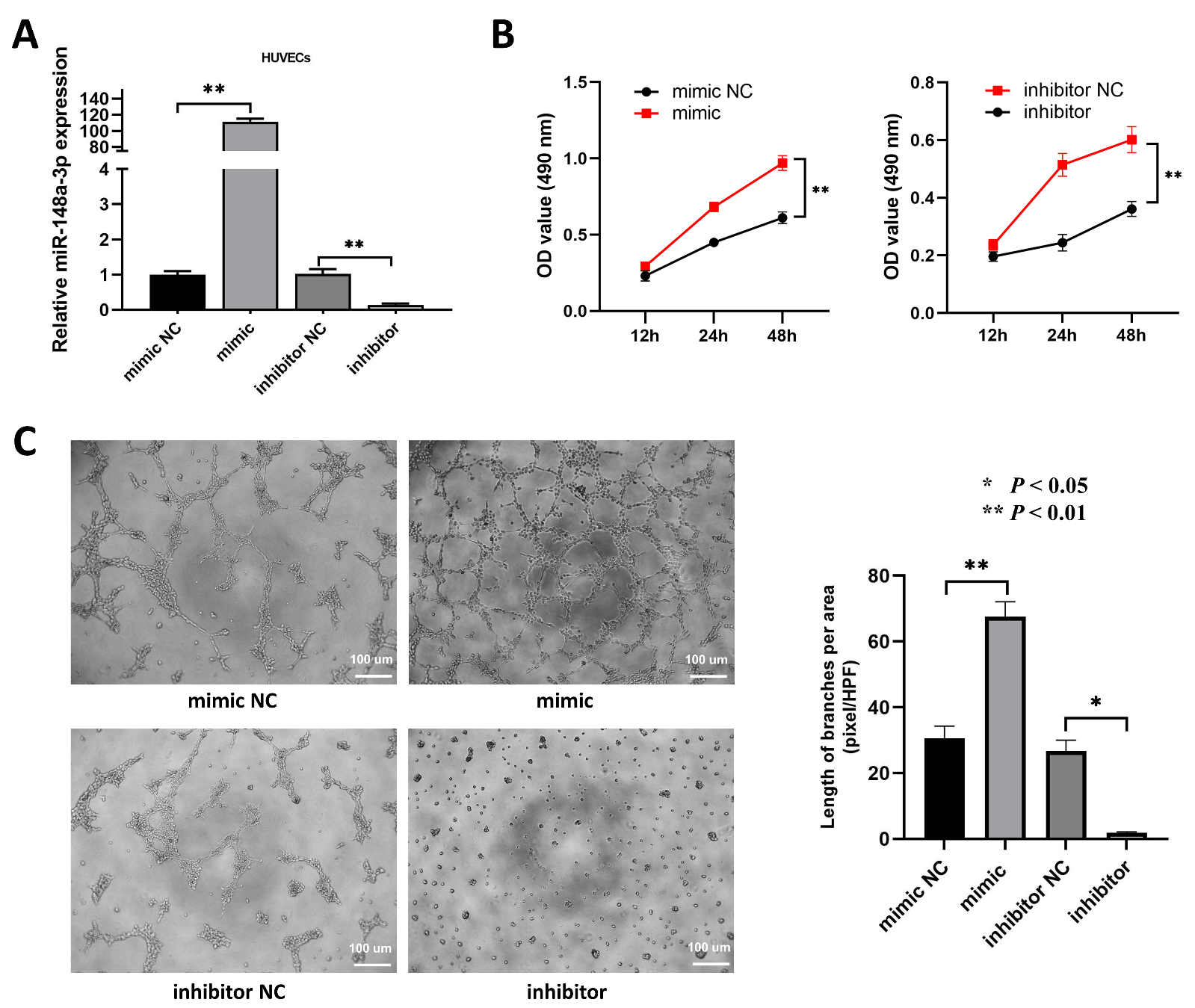


**Fig. S8. miR-148a-3p promotes proliferation and angiogenesis in HMVECs. (A)** Expression of miR-148a-3p was detected by qPCR in HMVECs transfected with miR-148a-3p mimic or inhibitor. **(B)** MTS assay to test proliferation ability of HMVECs transfected with miR-148a-3p mimic or inhibitor. **(C)** The angiogenesis capacity of HMVECs transfected with miR-148a-3p mimic or inhibitor was evaluated by tube formation assay. All data are shown as the means ± SEM. Statistical significance was analyzed by independent samples *t* test or one-way ANOVA followed by a Tukey *post hoc* analysis between two or multiple groups, respectively. **P* < 0.05, ***P* < 0.01. Scale bars, 100 um.

**Table S1.** Full gene names for pig cytokines and chemokines.

| Gene Abbreviation | Description |
| --- | --- |
| CCL2 | Chemokine (C-C motif) ligand 2 |
| CCL3L1 | Chemokine (C-C motif) ligand 3-like 1 |
| CTGF | Connective tissue growth factor |
| EGF | Epidermal growth factor |
| VEGFA | Vascular endothelial growth factor A |
| CXCL10 | C-X-C motif chemokine ligand 10 |
| IL1A | Interleukin 1, alpha |
| IL1B | Interleukin 1, beta |
| IL4 | Interleukin 4 |
| IL5 | Interleukin 5 |
| IL6 | Interleukin 6 |
| IL8 | Interleukin 8 |
| IL-10 | Interleukin 10 |
| IL13 | Interleukin 13 |
| TGFB1 | Transforming growth factor beta 1 |
| TGFB2 | Transforming growth factor beta 2 |
| TGFB3 | Transforming growth factor, beta 3 |
| TNF | Tumor necrosis factor |

**Table S2.** Full name of genes related to fibrosis.

| **Gene Abbreviation** | **Description** |
| --- | --- |
| ABL1 | C-abl oncogene 1, non-receptor tyrosine kinase |
| ACTA2 | Actin, alpha 2, smooth muscle, aorta |
| AGT | Angiotensinogen-like |
| AKT1 | V-akt murine thymoma viral oncogene homolog 1 |
| BCL2 | B-cell CLL/lymphoma 2 |
| Bmp7 | Bone morphogenetic protein 7 |
| CAV1 | Caveolin 1, caveolae protein, 22kDa |
| CCL11 | CCL11 |
| CCL2 | Chemokine (C-C motif) ligand 2 |
| CCL3L1 | Chemokine (C-C motif) ligand 3-like 1 |
| CTGF | Connective tissue growth factor |
| CCR2 | Chemokine (C-C motif) receptor 2 |
| CCR7 | Chemokine (C-C motif) receptor 7 |
| CEBPB | CCAAT/enhancer binding protein (C/EBP), beta |
| JUN | C-JUN protein |
| COL1A2 | Collagen, type I, alpha 2 |
| COL3A1 | Collagen, type III, alpha 1 |
| CREBBP | CREB binding protein |
| CXCR4 | Chemokine (C-X-C motif) receptor 4 |
| DCN | Decorin |
| EDN1 | Endothelin 1 |
| EGF | Epidermal growth factor |
| ENG | Endoglin |
| FASLG | Fas ligand (TNF superfamily, member 6) |
| GREM1 | Gremlin 1, DAN family BMP antagonist |
| HGF | Hepatocyte growth factor-like |
| IFNG | Interferon-gamma |
| IL10 | Interleukin 10 |
| IL13 | Interleukin 13 |
| IL13RA2 | Interleukin 13 receptor, alpha 2 |
| IL1A | Interleukin 1, alpha |
| IL1B | Interleukin 1 beta |
| IL4 | Interleukin 4 |
| IL5 | Interleukin 5 |
| ILK | Integrin-linked kinase |
| INHBE | Inhibin beta E chain-like |
| ITGA1 | Integrin alpha-1-like |
| ITGA2 | Integrin, alpha 2 (CD49B, alpha 2 subunit of VLA-2 receptor) |
| LOC100517053 | Integrin, alpha 3 (antigen CD49C, alpha 3 subunit of VLA-3 receptor) |
| ITGAV | Integrin, alpha V (vitronectin receptor, alpha polypeptide, antigen CD51) |
| ITGB1 | Integrin, beta 1 (fibronectin receptor, beta polypeptide, antigen CD29 includes MDF2, MSK12) |
| ITGB3 | Integrin, beta 3 (platelet glycoprotein IIIa, antigen CD61) |
| ITGB5 | Integrin, beta 5 |
| ITGB6 | Integrin, beta 6 |
| ITGB8 | Integrin, beta 8 |
| LOC100038019 | Transforming growth factor beta receptor II |
| THBS2 | Thrombospondin-2-like |
| SNAI1 | SNAIL-like zinc finger protein |
| LOX | Lysyl oxidase |
| LOC100627243 | Latent transforming growth factor beta binding protein 1 |
| MMP1 | Matrix metallopeptidase 1 (interstitial collagenase) |
| MMP13 | Matrix metalloproteinase 13 precursor |
| MMP14 | Matrix metallopeptidase 14 (membrane-inserted) |
| MMP2 | Matrix metallopeptidase 2 (gelatinase A, 72kDa gelatinase, 72kDa type IV collagenase) |
| MMP3 | Matrix metallopeptidase 3 (stromelysin 1, progelatinase) |
| MMP8 | Matrix metallopeptidase 8 (neutrophil collagenase) |
| MMP9 | Matrix metallopeptidase 9 (gelatinase B, 92kDa gelatinase, 92kDa type IV collagenase) |
| MYC | V-myc myelocytomatosis viral oncogene homolog (avian) |
| NFKB1 | Nuclear factor of kappa light polypeptide gene enhancer in B-cells 1 |
| PDGFA | Platelet-derived growth factor alpha polypeptide |
| PLAT | Plasminogen activator, tissue |
| PLAU | Plasminogen activator, urokinase |
| PLG | Plasminogen |
| SERPINA1 | Serpin peptidase inhibitor, clade A (alpha-1 antiproteinase, antitrypsin), member 1 |
| SERPINE1 | Serpin peptidase inhibitor, clade E (nexin, plasminogen activator inhibitor type 1), member 1 |
| SERPINH1 | Serpin peptidase inhibitor, clade H (heat shock protein 47), member 1, (collagen binding protein 1) |
| SMAD2 | SMAD family member 2 |
| SMAD3 | SMAD family member 3 |
| SMAD4 | SMAD family member 4 |
| SMAD6 | SMAD family member 6 |
| SMAD7 | SMAD family member 7 |
| SP1 | Sp1 transcription factor |
| STAT1 | Signal transducer and activator of transcription 1, 91kDa |
| TGFB1 | Transforming growth factor, beta 1 |
| TGFB2 | Transforming growth factor, beta 2 |
| TGFB3 | Transforming growth factor, beta 3 |
| TGIF1 | TGFB-induced factor homeobox 1 |
| THBS1 | Thrombospondin 1 |
| TIMP1 | TIMP metallopeptidase inhibitor 1 |
| TIMP2 | TIMP metallopeptidase inhibitor 2 |
| TIMP3 | Tissue inhibitor of metalloproteinase-3 |
| TIMP4 | Tissue inhibitor of metalloproteinase-4 |
| TNF | Tumor necrosis factor |
| VEGFA | Vascular endothelial growth factor A |
| ACTG1 | Actin gamma 1 |
| B2M | Beta-2-microglobulin |
| GAPDH | Glyceraldehyde-3-phosphate dehydrogenase |
| HPRT1 | Hypoxanthine phosphoribosyltransferase 1 |
| RPL13A | Ribosomal protein L13a |
